# A systems level approach to study metabolic networks in prokaryotes with the aromatic amino acid biosynthesis pathway

**DOI:** 10.3389/fgene.2022.1084727

**Published:** 2023-01-16

**Authors:** Priya V. K, Somdatta Sinha

**Affiliations:** ^1^ National Institute of Technology Calicut, Kattangal, Kerala, India; ^2^ Indian Institute of Science Education and Research Kolkata, Mohanpur, West Bengal, India

**Keywords:** metabolic pathways, aromatic amino acids biosynthesis, network analysis, flux balance analysis (FBA), systems biology

## Abstract

Metabolism of an organism underlies its phenotype, which depends on many factors, such as the genetic makeup, habitat, and stresses to which it is exposed. This is particularly important for the prokaryotes, which undergo significant vertical and horizontal gene transfers. In this study we have used the energy-intensive Aromatic Amino Acid (Tryptophan, Tyrosine and Phenylalanine, TTP) biosynthesis pathway, in a large number of prokaryotes, as a model system to query the different levels of organization of metabolism in the whole intracellular biochemical network, and to understand how perturbations, such as mutations, affects the metabolic flux through the pathway - in isolation and in the context of other pathways connected to it. Using an agglomerative approach involving complex network analysis and Flux Balance Analyses (FBA), of the Tryptophan, Tyrosine and Phenylalanine and other pathways connected to it, we identify several novel results. Using the reaction network analysis and Flux Balance Analyses of the Tryptophan, Tyrosine and Phenylalanine and the genome-scale reconstructed metabolic pathways, many common hubs between the connected networks and the whole genome network are identified. The results show that the connected pathway network can act as a proxy for the whole genome network in Prokaryotes. This systems level analysis also points towards designing functional smaller synthetic pathways based on the reaction network and Flux Balance Analyses analysis.

## 1 Introduction

Biochemical pathways in cells underlie cellular functions, and hence its phenotype. These are regulated by many direct and indirect, and hardwired and transient factors. Evolution of multi-step biochemical pathways in any species depends upon how natural selection shapes the evolution of a set of enzyme-coding genes catalysing the constituent chemical reactions, such that the required end-product is made (Flowers et al., 2007; Invergo et al., 2013). However, the genes, enzyme and pathways do not function independently. In each species, they exist in the context of a large biochemical network, consisting of other genes, enzymes and pathways interacting with each other, and with the intra- and extra-cellular environments. Hence in order to understand the interactions and effects in functionally related pathways, we need to study the properties of subsets of metabolic networks at different levels.

To study how pathways regulate their function with respect to each other, we chose the highly branched aromatic amino acid (Tryptophan-Tyrosine-Phenylalanine, TTP) biosynthesis pathway as an example. This pathway is responsible for the production of three aromatic amino acids; Tryptophan, Tyrosine and Phenylalanine–all requiring high energy for their synthesis. The TTP pathway has been studied previously for its role in the production of secondary metabolites (Herrmann 1995; Herrmann and Weaver 1999), and its usage as target for several antibiotics, fungicides and herbicides (Roberts et al., 2002; Abell et al., 2005; Webby et al., 2005). The TTP pathway is present in most of the prokaryotes, but is lost in higher eukaryotes and mammals (Xie et al., 2003), thus requiring higher organisms to get some of these amino acids as food additives. Even in the TTP prototrophs, the evolutionary history of the pathway is convoluted due to instances of horizontal gene transfer and is characterized by many isozymes, bi-functional enzymes and gene fusions (Bentley and Haslam 1990; Xie et al., 2003; Richards et al., 2006; Priya et al., 2014).

Traditionally, specific pathways such as, the Tryptophan biosynthetic pathway, have been studied in depth both experimentally and theoretically using mathematical models (Yanofsky et al., 1987; Sinha 1988; Santillan and Mackey 2001; Castro-López et al., 2022). However, in the post-genomic era, most of the studies have focussed on network modelling and analysis of the whole cellular metabolism (Fairlamb 2002; Ma and Zeng 2003; Gerlee et al., 2009). In recent times, the principles of Systems Biology have been used extensively to study metabolic pathways at different scales (Nielsen 2017), and reconstruction of whole genome metabolic networks from their genome sequences has been an active area of study (Khodayari et al., 2016; Norsigian et al., 2018; Bagheri et al., 2019).

From the perspective of the intracellular biochemical network, the maze of neighbouring pathways, that are connected through sharing one or more metabolites, can influence the function and evolution of each other. Yet, study of pathways *in the context of each other* is rarely done across species. Hence in order to study the contextual influence of the inter-connected pathways, we use complex network analysis on the TTP pathway reactions network in 29 Bacteria and Archaea. Several FBA and network models have shown how various reactions are connected and used smaller subsystems to improve production or for finding new drug targets. But in these networks, the pathways present in one particular organism were studied, for example the network for disease associated pathway cluster for Huntington disease (Kakouri et al., 2019) or the network of interacting pathways to find drug targets (Raman et al., 2005; Chen et al., 2016). Our study is different from these since we are using data from 29 species of free-living Bacteria and Archaea from diverse environments and metabolic activities and we have formed a network of pathways that are connected to the TTP pathway that is common across the 29 species. This is a novel method to understand how the pathways are interconnected and function in context to each other. We have assessed the variations in the topological properties of the TTP reaction network nodes after adding the neighbouring pathways, in the combined reaction networks. Our results show the contextual variations of the topological properties of the TTP reaction network nodes in the combined network, and study their similarity across bacteria and archaea.

Network representation and analysis of metabolic pathways offers a convenient and useful mode for understanding the role of the connectivity patterns of the reaction nodes in interconnected pathways. However, the chemical reactions at each step decide the function of the pathway. Flux Balance Analysis (FBA), a constraint-based approach to model organisms based on mass-energy balance, and flux limitations (Kauffman et al., 2003) are used to understand how the reaction product flux functioned in the pathway. The FBA has been used previously for representing and modeling the growth of many organisms such as, *E. coli* (Edwards and Palsson 2000; Burgard and Maranas 2001), *L. lactis* (Flahaut et al., 2013), *S. coelicolor* A3(2) (Borodina et al., 2005), *G. oxydans* (Wu et al., 2014), *etc.* We used the FBA to study the effect of mutation or deletion of genes/reactions, present in the TTP pathway and other connected pathways - on the flux through the TTP pathway. This study yielded information on those reaction steps that have a direct effect on the production of aromatic amino acids, in the context of the larger reaction network. Comparing the network and FBA analysis results, we show that, at the systems level, the pathway activities are dependent on a smaller set of reactions that are important for its biochemical activities. This also indicates that a smaller reaction network of the important reactions and enzymes may be chemically engineered for a functional pathway instead of the existing whole metabolic pathway that has evolved through a step-by-step evolutionary historical contingency.

## 2 Results

**FIGURE 1:**
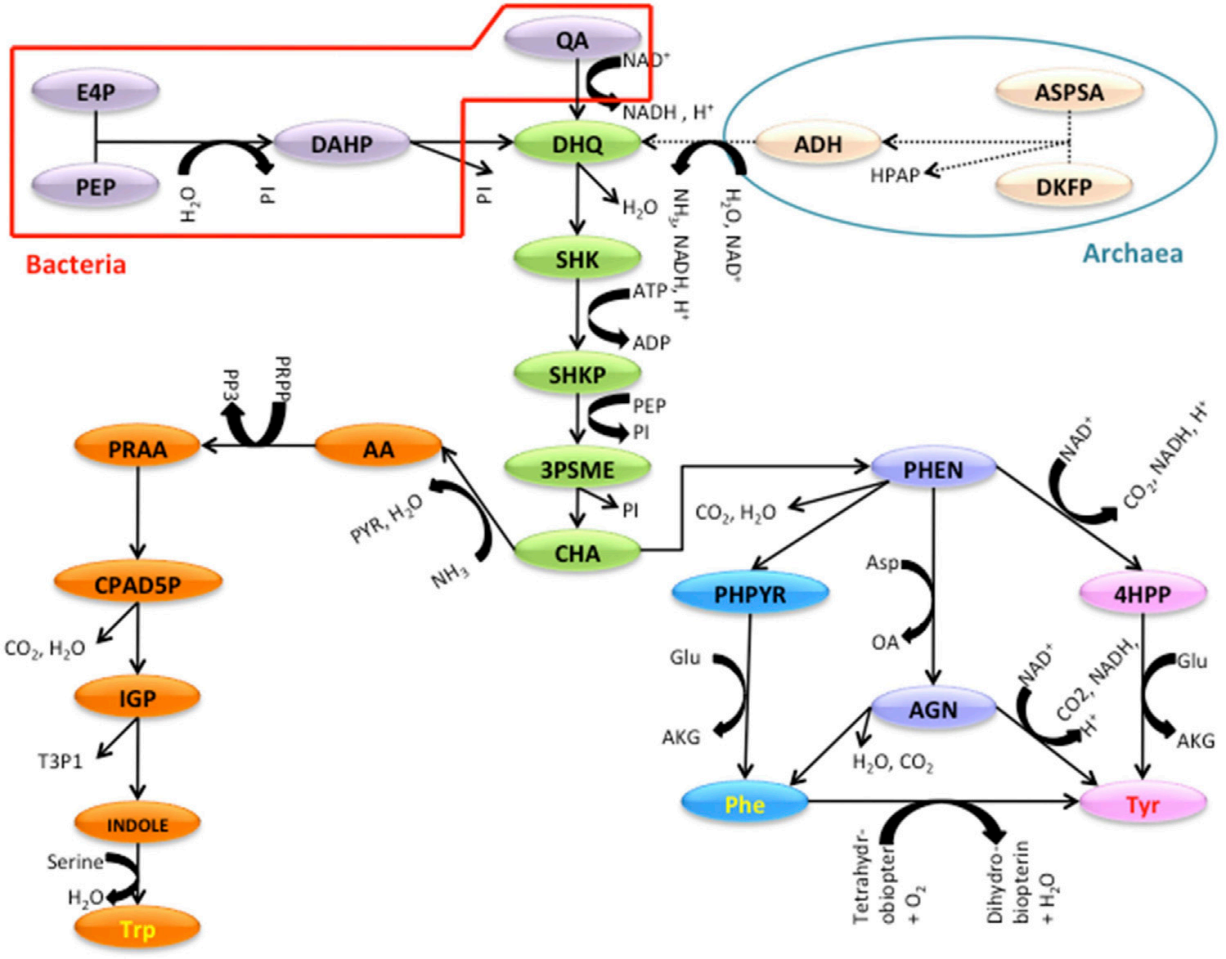
The TTP Pathway studied with the sections indicated. Red and blue shapes are specific to Bacterial and Archaeal TTP pathway. The Green substrates are part of the Shikimate section. Orange is for Tryptophan and Blue and Pink are for the Phenylalanine and Tyrosine parts. INPUT SECTION: In bacteria; E4P (D-Erythrose-4-phosphate), PEP (Phosphoenol pyruvate), DAHP (2-Dehydro-3-deoxy-D-arabino-heptonate 7-phosphate), QA (Quinate), DKFP (6-deoxy-5-ketofructose-1-phosphate). In archaea; ASPSA (Aspartate semi aldehyde). SHIKIMATE SECTION: DHQ (3-dehydroquinate), SHK (Shikimate), SHKP (Shikimate 3-phosphate), 3PSME (5-O-(1-Carboxyvinyl)-3-phosphoshikimate), CHA (Chorismate). TRYPTOPHAN SECTION: AA (Anthranilate), PRAA (N-(5-Phospho-D-ribosyl)anthranilate), CPAD5P (1-(2-Carboxyphenylamino)-1-deoxy-D-ribulose 5-phosphate), IGP (Indoleglycerol phosphate), INDOLE, Trp (Tryptophan). PHENYLALANINE AND TYROSINE SECTION: PHEN (Prephenate), PHPYR (Phenylpyruvate), 4HPP (4-Hydroxyphenylpyruvate), AGN (L-Arogenate), Phe (Phenylalanine), Tyr (Tyrosine).

***The TTP Pathway*:** A reconstructed common TTP pathway model for Bacteria and Archaea is shown in Figure 1. The pathway is divided into four sections (see Figure 1 legend) where the additional reactions specific to bacteria are shown in red and that for Archaea in blue boundaries at the top.

### 2.1 Network analysis

The directed reaction networks were constructed for the TTP pathway for 29 organisms (Supplementary Table S1), and their network properties such as Degree, Clustering Coefficient, Closeness Centrality and Betweenness Centrality were studied.

#### 2.1.1 TTP pathway network and its network properties

**FIGURE 2:**
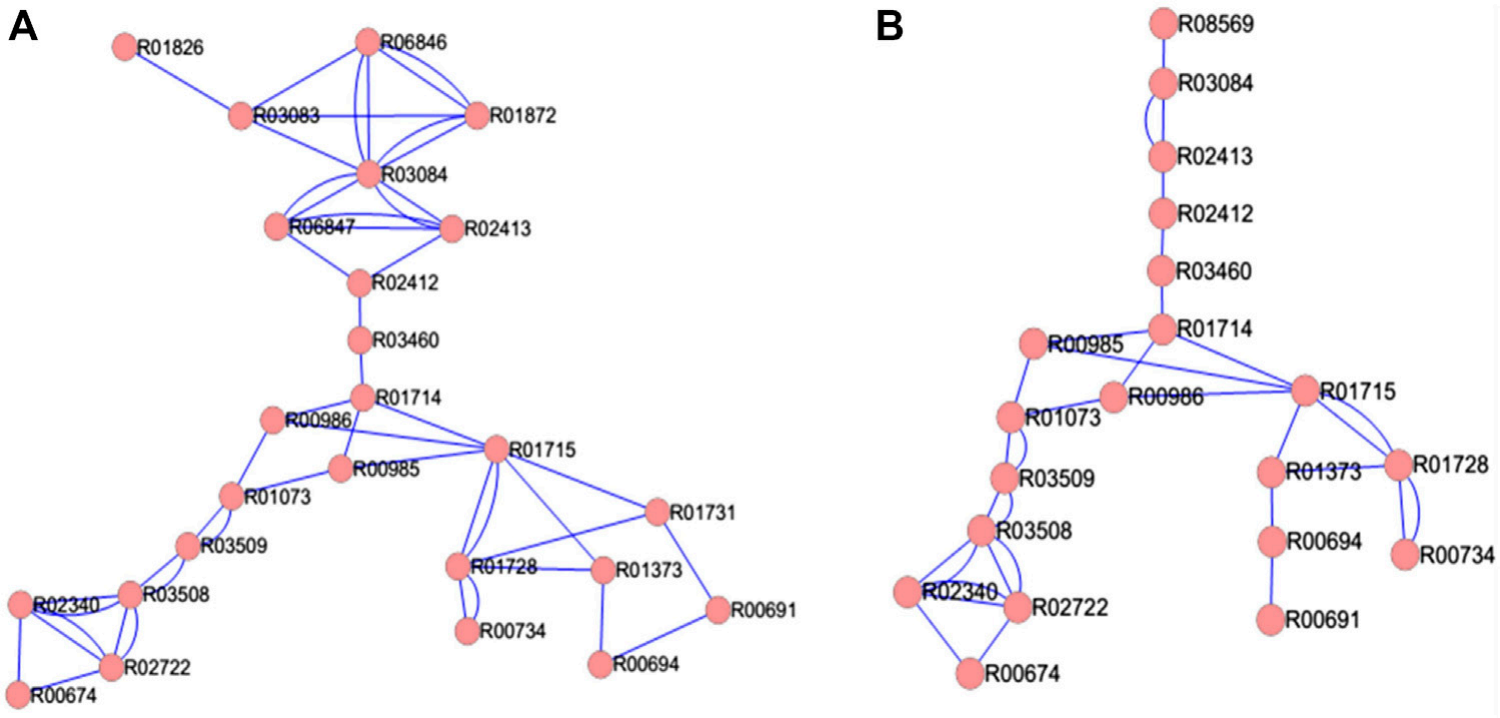
TTP network in **(A)** bacteria *E. coli* and **(B)** Archaea *N. pharaonis*. The double edges indicate reversible reactions.

The TTP reaction pathway is a linear network (Figure 2) with very few connections other than due to consecutive dependence. The Archaeal and Bacterial TTP networks are topologically similar. Figure 2 shows *E. coli* and *N. pharaonis* TTP networks as examples for the Bacterial and Archaeal organisms. The major difference between the two networks lies in the input region, since in many Archaea the DKFP pathway provides the precursors for the formation of 3-dehydroquinate, whereas in Bacteria it is from the Pentose phosphate pathway and Glycolysis (see Figure 1). The number of reaction nodes and edges varies among both bacterial and archaeal species. For example, in Bacteria, the reaction nodes vary between 18 (for *S. thermophilus*) and 25 (for *E. coli*), and the number of edges between 30 (*S. thermophilus*) and 50 (*E. coli*).

The difference in the number of nodes between the organisms is because there are multiple reactions that provide different paths for the production of the same metabolite. Due to this, the number of connected pathways also differs across the organisms under study. For further analysis, only those reactions and pathways are chosen that are common across all the 29 organisms (Supplementary Table S2 and S3).

**FIGURE 3:**
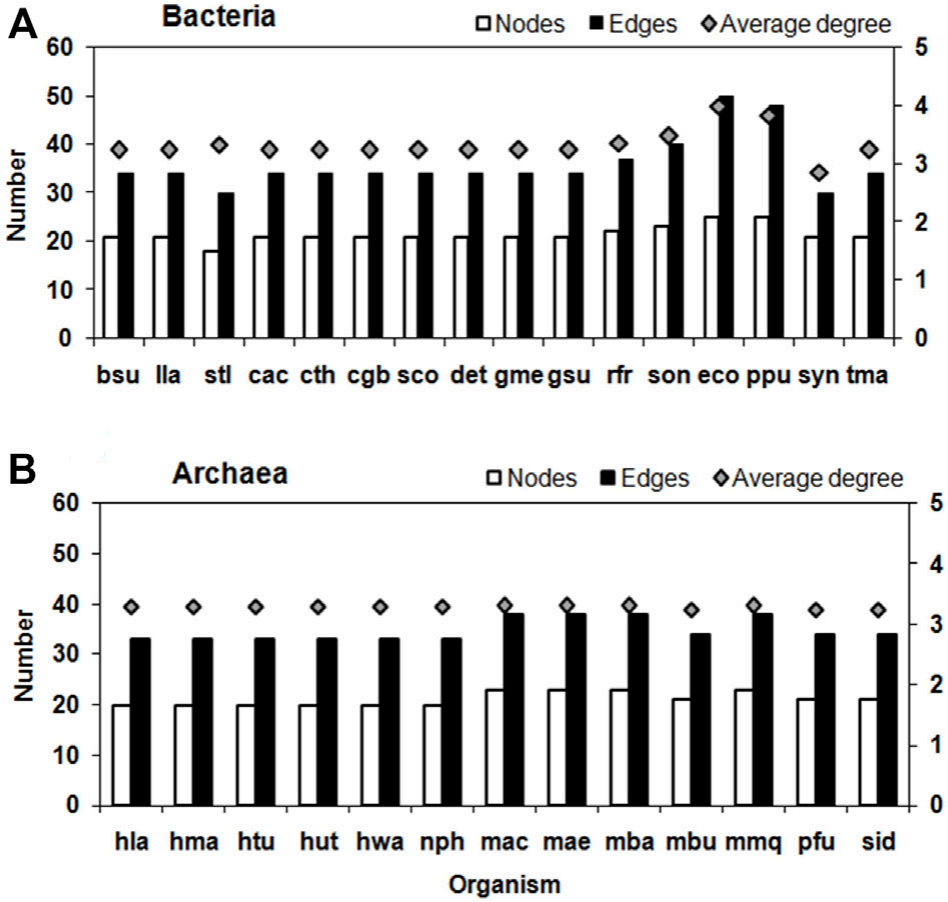
Number of Nodes, Edges (left *Y*-axis) and average degree (right *Y*-axis) of TTP pathway network in **(A)** Bacteria and **(B)** Archaea. See Supplementary Table S1 for the three lettered species names.

The average Degree in these TTP networks is between 2.86 (*Synechocystis sp*. PCC 6803) and 4 (*E. coli*), which further shows how sparsely connected the network is. Based on these properties, the Bacteria and Archaea networks do not differ much. Amongst the Bacteria, the Proteobacteria tend to have higher number of nodes and edges. The Gamma-proteobacteria, *E. coli* and *P. putida* has the highest number of nodes, edges and average degree for their TTP pathway network (Figure 3).

***Connected Pathway of TTP*:** A connected pathway is one in which at least one reaction of that pathway either produces or consumes a metabolite that is either consumed or produced by the TTP pathway. Even though there is a slight difference between the bacterial and the archaeal TTP pathway, the entire metabolic network of these organisms may differ greatly from each other. This may cause the pathways associated with the TTP pathway to differ between organisms. Therefore, only the reactions and associated pathways that are common among all the 29 organisms under study are discussed here (Supplementary Table S2 and S3).

#### 2.1.2 Network properties of connected pathway networks

The average network properties of the connected networks, i.e., the TTP network combined with each of the connected networks (as given in Supplementary Tables S2 and S3), were calculated for all organisms. First the global properties of the connected pathway networks are given, and then local node-level properties are discussed.

Global properties of connected networks

***Nodes***: The number of nodes of the combined pathways are significantly different from their TTP network in all the Bacteria and Archaea (Wilcoxon test, *p*-value <.05) (Supplementary Figure S1). The highest number of nodes is in Microbial metabolism in “diverse environments” (map01120), Biosynthesis of Amino Acids (map01230), Purine metabolism (map00230) and Carbon metabolism (map01200). Except for the 2-Oxocarboxylic acid metabolism (map01210) and Methane metabolism networks (map00680) all the other connected networks of Archaea have lower number of nodes than its Bacterial counterpart. In Bacteria, the highest variation in the number of nodes is in map00330, map01120, map00230 and map00240.

Bacterial networks show larger variation (std dev range: 1.67–16.18) in node numbers than Archaeal networks (std dev range: 1.34–8.49), and the main contributor to this are the Proteobacteria. Except for TTP, and the other 7 out of 17 connected pathways (e.g., map00340, map01230, map00020, map01200, map00010 and map00260), the rest of the connected networks differ significantly between Bacteria and Archaea (Wilcoxon test, *p*-value <.05). Bacterial networks have significantly higher number of nodes compared to the Archaeal networks in few pathways, but in map01210 and map00680 they are significantly more in Archaea (Wilcoxon test, *p*-value <.05).

***Edges:*** A similar distribution is seen in the edge numbers and degree in both Bacteria and Archaea (Supplementary Figure S2 and S3). In Archaea, for example, the Glycolysis pathway adds a higher number of edges than in Bacteria, suggesting larger number of connections between the nodes in Archaea. The addition of connected pathways significantly changes the degree in all the pathways, except map00330 and map00051 in Bacteria, and map00970 and map00051 in Archaea. Here also the variation is more in Bacteria than in Archaea. Contrary to all the other properties, the variation in the degree is slightly more in Archaea (std. dev. range: .03–.99) than in Bacteria (std. dev. range: .1–.87), and significant differences are observed in map00970, map01120, map01210, map00020, map00010, map00680, map00230, map00240 and map00030 between Bacteria and Archaea (Wilcoxon test, *p*-value <.05). Furthermore, addition of sparsely connected map00340, map00970 and map00270 decreases the average degree of the combined networks (Supplementary Figure S3).

***Average Path Length***: Addition of new nodes to the existing TTP pathway does not always increase the average path length of the network proportionately (Supplementary Figure S4), except for the addition of map00020 in Bacteria and map01210, map00020, map00010, map00260, map00270 and map00051 in Archaea. For all other pathways, the addition significantly changes the Average Path Length (APL) of the network (Wilcoxon test, *p*-value<.05). There are significant differences in the APL between Bacteria and Archaea in map01120, map01210, map01230, map00010, map00680, map00240 and map00030.

These results indicate that as the metabolic networks expand, due to addition of nodes, the network properties do not change proportionately–they depend on the connection point to the TTP pathway, and the topology of the added pathway. They also differ between and within Bacterial and Archaeal species for the same connected pathway even though the basic TTP pathway do not differ much between the two types of Prokaryotes.

Local properties of connected networks

The addition of the *connected* networks to TTP pathway network not only changes the global properties of the combined pathway networks, but also the properties of the individual TTP network nodes.

***Degree*:** The comparison of ***Degree*** across the different connected networks show that there is no consistent difference between Bacterial and Archaeal networks. The 3 out of 15 common reactions showed no variations in degree, while 5 out of 15 have significantly different Degree in connected networks (>2 std dev) across different organisms in the connected pathways. Addition of certain pathways such as the map01230 introduces fairly large variations in the Degree for *E. coli*. (shown in Supplementary Figure S5), and *C. glutamicum*, *C. acetobutylicum*, *M. barkeri*, *R. perfringens* in the TTP reaction network nodes. The same in *H. turkmenica* show the least variability in their degree across all pathways. This result, interestingly, clearly demonstrates that individual reactions change their connectivity pattern on addition of pathways, and this is not necessarily due to direct attachment of the connecting pathway to that node. It could also be due to changes in their biochemical interactions facilitated due to the new pathway topology in different organisms.

***Clustering Coefficient*** (***CC*):** The CC of the TTP pathway reactions also change due to the addition of the connected networks (see Supplementary Figure S6). Although, out of 17 connected pathways, the CC of 4 remain the same, but 3 show significant differences (>2 std dev). These are for the addition of map01230, map00230, and map01120 as the addition of new nodes in these networks reduces the CC of these nodes. Bacteria and Archaea show similar variation in their CC. A summary of changes in Degree and Clustering Coefficient in TTP nodes are shown in Table 1.

***Closeness centrality*:** Addition of pathways tends to change the path length, which is reflected in the parameter Closeness centrality. The TTP pathway has a high Closeness centrality, and the addition of other pathways increase the number of Nodes and the Closeness centrality of the overall network. Analysis showed that most of the connected networks, with the exception of map00970, have a significantly different Closeness centrality when compared to the isolated TTP pathway. Addition of map01230 increases Closeness centrality while addition of map00250, map00330, map01120, map01210, map01200, map00260, map00230 - decreases it for the TTP nodes. The pathways, such as, map01120 and map01200 have varying results in different organisms due to the diverse environments in which these organisms survive. This increase and decrease in the network parameter Closeness centrality indicates that the local network properties of the TTP pathway reactions nodes can change in a non-consistent manner even when the network is expanding due to the addition of nodes (see Supplementary Figure S7).

***Betweenness centrality*** (***BC***): BC of a node is an important property, as it signifies the central position of the node in the network in terms of transfer of information from all other nodes. There is a general decrease in this network parameter for most connected pathways across all TTP nodes. However, the addition of map01230 and map00970 also significantly alter the BC of the common reactions (z-score >3) across all organisms. R00674 show *almost no* variation in its BC among the combined pathways of different organisms, since it is at the terminal end of the network. The analysis of the change in BC in TTP nodes showed that, across organisms, most of the variation is observed in the map00030. The addition of this pathway to TTP changes the topology of the combined network in such a manner that it induces changes in the BC in several nodes. The reaction node R01073 in TTP pathway shows considerable increase in BC on addition of map00030 and map00340 due to the addition of pathways that are linear. BC of the terminal reactions, such as R00674 and R02722 in the TTP pathway, increases significantly due to the addition of the connected pathways which occur in very few cases. BC of a node being an important property in terms of transfer of information from all other nodes, our results show that only those pathways change the BC of the TTP nodes, which change the topology of the combined network based on where the added pathway is connected to the TTP network (Supplementary Figure S8).

**TABLE 1:** Changes in Degree and Clustering Coefficient in nodes.

	Degree	Clustering coefficient
No significant variation	R03508	R03460
	R03509	R03508
	R02340	R03509
		R02340
		R02722
Significant variation (Std. dev. >2)	R02722	R01073
	R01073	
	R03460	R01373
	R01714	
	R00674	R01714
	R01073	

#### 2.1.3 Combined Connected Network (CCN) of TTP

**FIGURE 4:**
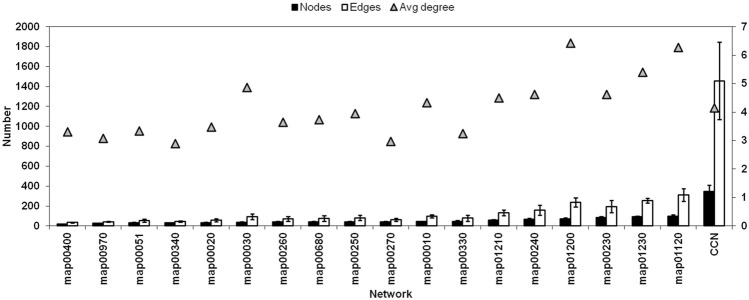
Network size (number of Nodes), Number of Edges, and the Average Degree of each connected networks and the CCN (Refer Supplementary Table S2 for pathway names).

Till now the network properties of the TTP pathway network, in combination with each of the connected pathways (as in Supplementary Table S2 and S3), have been studied. The *Combined Connected Network* (CCN) is the combined network of the TTP pathway with all the connected pathways added together. It gives an integrated view of the TTP pathway embedded in the metabolic network of the 17 reaction pathways directly connected to it for each of the organisms under study. The question addressed here is how the network properties of the individual nodes of the TTP pathway network change in such a combined network, because of the change in the topology and connectivity patterns in the CCN. Combining the connected pathway networks to TTP added new nodes and edges to the TTP pathway. As will be shown below, some of these additions significantly change the topological properties of the TTP pathway reactions (Table 2). For example, the TTP reaction node R02722 is the only one that shows increase in Betweenness Centrality in the CCN. This is due to the addition of the highly interconnected *Glycine*, Serine and Threonine pathway in the CCN through that node. The addition of the highly interconnected pathways, such as the amino acid biosynthesis pathway, or addition of a few nodes, as in the case of map00970, could significantly alter the properties of the TTP nodes (Figure 4).

**TABLE 2:** Average Betweenness and average Closeness values (for 29 organisms) for the nodes in the TTP pathway - in isolation and in the Combined Connected Network (CCN). The standard deviations are not shown as the values are very low.

	Betweenness		Closeness	
	TTP	CCN	TTP	CCN
R02412	.156	.007	.012	1.16 × 10−05
R03460	.183	.008	.014	1.16 × 10−05
R01373	.084	.007	.013	1.16 × 10−05
R01714	.205	.009	.016	1.17 × 10−05
R01715	.18	.017	.016	1.17 × 10−05
R00674	0	0	.008	1.17 × 10−05
R02722	.011	.047	.009	1.17 × 10−05
R02340	.05	.001	.009	1.17 × 10−05
R03508	.15	.045	.011	1.17 × 10−05
R03509	.178	.045	.012	1.17 × 10−05
R01073	.196	.065	.014	1.17 × 10−05
R00985	.102	.015	.016	1.17 × 10−05
R00986	.102	.015	.016	1.17 × 10−05
R03084	.085	.006	.009	1.16 × 10−05
R02413	.116	.006	.01	1.16 × 10−05

**FIGURE 5:**
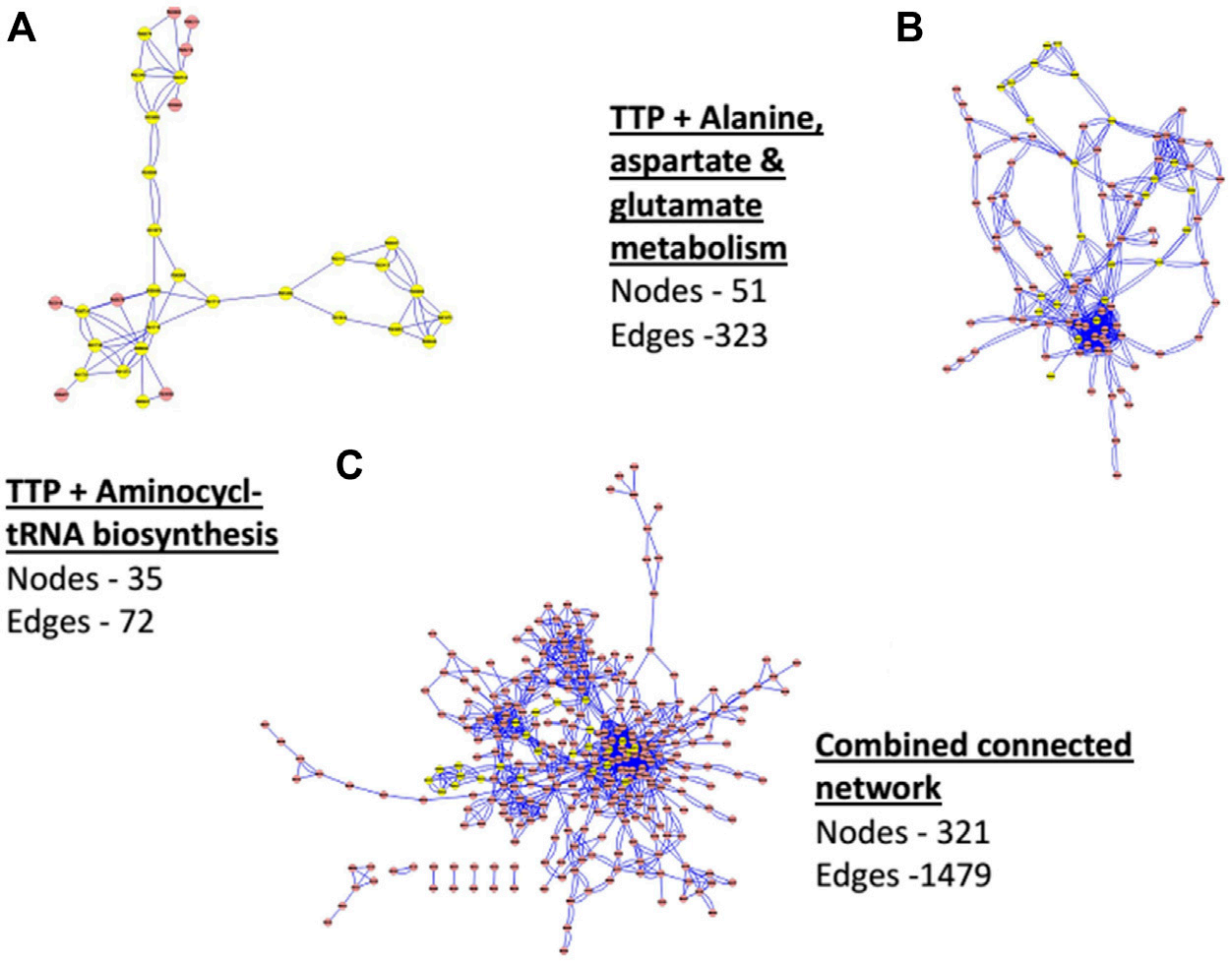
Network of TTP (with Yellow Nodes) with connected networks of **(A)** Aminoacyl tRNA biosynthesis pathway (map00970), **(B)** Alanine, Aspartate and Glutamate metabolism (map00250) and **(C)** Combined Connected Network (CCN).

Figure 4 shows the comparison of a few network properties among each connected pathway in all organisms (see Supplementary Table S2 for pathway names) and the CCN. Network size (number of Nodes), total number of Edges, and the average Degree of each connected networks are shown along with that of the CCN. Figure 5 shows the topology of the TTP reaction network (Yellow nodes) when connected with A) Aminoacyl t-RNA biosynthesis pathway (map00970), B) Alanine, Aspartate and Glutamate metabolism (map00250), and C) Combined Connected Network (CCN). It is clear that increasing the number of Nodes does not necessarily increase the average Degree of the network (Figure 4; Figure 5).

For CCN, the average degree is 4.15 - quite low even though the average network size is large (321 Nodes). The number of Edges, though not additive, is also quite large (1479). This indicates, as is seen in Figures 5A–C also, that the CCN has a topology that is largely branched with many linear sections. The TTP pathway is largely linear and is a non-redundant network. Hence most of the nodes are equally important for the pathway to function, even though each of them has different network properties (as mentioned in the previous sections). Addition of other pathways can cause nodes to change their local network properties. Low Centrality measures point towards the fact that the CCN has a non-compact topology with large linear sections. This is due to the underlying chemical basis of the network, where substrate-product reactions are quite specific to their chemical nature, and the same chemical species cannot be obtained through different chemical reactions.

The “hubs” of the network parameters - Degree, Betweenness Centrality and Closeness Centrality - are reaction nodes in the network with the highest value of the respective parameters. We use a cut off for selecting Hubs as the “Nodes in top 20%” of each of the measures. The CCN have few Degree hubs, since these networks are characterized by a large number of Nodes with low degrees, and very small number of Nodes with high degrees, and non-redundant routes for metabolism. There are 66 reaction nodes in the CCN that are found as hubs common to all organisms. Most of them are either Betweenness Centrality or Closeness Centrality hubs (Supplementary Table S4). Since these hub reactions in the CCN are important for the network, these might also be important for the functioning of the TTP pathway in the integrated network. It is clear that many (9 of 15) of the TTP reactions have now increased their Betweenness Centrality and Closeness Centrality when in the context of other connected pathways. The nature of these networks is generally linear sequence of chemical reactions leading to formation of specific products. However, these specific reaction pathways interact to facilitate cross-talk to promote coordinated response of the cell. Therefore, increasing the centrality measures seems to be a functionally suitable strategy, since increasing degree may not be chemically possible. The changed network parameters of the TTP nodes in the combined network (CCN) points towards their role in changing/modifying their function when in context of other pathways. This can lead to change in their biochemical attributes (such as, reaction velocity, flux, regulation, *etc.*).

### 2.2 Flux balance analysis (FBA)

FBA is done in order to analyze the flux passing through the reaction steps of the TTP pathway during wild-type growth, and after perturbations (e.g., loss of reaction due to deletion mutation, or lowering of efficiency of the reaction), in order to understand the influence of different reactions on the working of the TTP pathway. The flux analysis (see Methods section), for the TTP pathway was done on the complete genome scale models of *E. coli* (Feist et al., 2007) and *M. barkeri* (Gonnerman et al., 2013). The genome scale *E. coli* model, considered here, consisted of a total of 2382 reactions, 1261 genes, and 1668 metabolites; and, the *M. barkeri* model consisted of 815 reactions, 750 genes, and 718 metabolites.

#### 2.2.1 Flux analysis in TTP pathway

Production of aromatic amino acids (TTP) in the cell is a high energy consuming process (Akashi and Gojobori 2002). This energy cost is reflected in their low usage in the polypeptide chain, and in the flux passing through the TTP pathway in almost all the organisms. All the flux mentioned here on will be in mmolgDW^−1^h^−1.^ The Tryptophan section has the least amount of flux passing through it: .0418 for *E. coli*, and .0013 for *M. barkeri*. The Phenylalanine section (.1296 for *E. coli* and .0041 for *M. barkeri*), and Tyrosine section (.1018 for *E. coli* and .0035 for *M. barkeri*) have higher fluxes (Supplementary Figure S9 and S10). The list of reactions present in *E. coli* and *M. barkeri* is given in Supplementary Table S5.

**Fluxes through the TTP pathway for *E. coli* and *M. Barkeri* are different**

1) In both the organisms, fluxes through the Input and Shikimate section are higher than the rest of the sections, because the flux is undivided in these sections. At Chorismate synthase reaction (CHORS), the flux is distributed between the two branches depending on the coefficients of Trp and Phe-Tyr in the biomass equation. All the flux passes through TRPAS2 of the Trp section in *E. coli.* In *M. barkeri*, it takes the reaction TRPS1 to produce the same metabolite Tryptophan.
2) Compared to Bacteria *E. coli,* the Archaea *M. barkeri* has a lower flux. It may be noted that the growth rate for *E. coli* is higher than that of *M. barkeri*, which also shows up in the differences in the media and biomass equations of the two organisms. Out of 1339 unique reactions (as mentioned in section 2.2 of CCN of *E. coli*) present in the whole FBA, deletion of 175 reactions was found to be adversely affecting the production of the aromatic amino acids. We reduced the efficiency of the *E. coli* TTP pathway genes to find the effect of such changes in the production of the aromatic amino acids. A 100% reduction (deletion) of the TTP pathway genes is shown in (Supplementary Figure S11; Supplementary Table S8). Deletion of genes in the TTP pathway leads to no flux through any of the reactions except for TRPAS2, TRPS1, TRPS2 and TRPS3 which are alternate pathways to each other. If the bounds of the flux of the reactions are reduced to 90% of the flux one by one through the reactions, then a marked reduction is seen in the flux through the network (Supplementary Table S9). Suggesting that even though the amount of flux passing through the reactions are very low, they still play a major role in the biomass formation of the organisms. For example, constraining the flux through PHETA1 to −.117 (reversible reaction) leads to a reduction of the flux through the Input and Shikimate section to .247 (.274 in Wild type-WT) and .038 through the tryptophan section (.042 in WT) and .117 in Phenylalanine (.13 in WT) and .092 through Tyrosine (.102 in WT) (Supplementary Figure S12).
In *M. barkeri*, out of the 815 reactions present in the FBA model, the deletion of 250 reactions shows adverse effect on the production of TTP. Many of these pathways are common between *E. coli* and *M. barkeri*, but some of them are unique to either Bacteria or Archaea, as the metabolism of these two organisms are different–in some cases. For example, the pathway for Glycerophospholipid biosynthesis pathway influences TTP production in *E. coli*, while the Methanofuran B biosynthesis and Methanogenesis pathways influences TTP production in Archaea *M. barkeri.*
3) Reducing single gene efficiency does not significantly affect TTP production in *M. barkeri* because there are alternative reactions for some reactions, which provide other routes for producing the same metabolite. This indicates that the TTP pathway is more robust in this organism in terms of random gene/reaction deletions. Deletion of genes involved in all reactions, except ANS, ANS2, TRPS1, TRPS2, TRPS3, leads to no flux through the TTP pathway (Supplementary Table S10; Supplementary Figure S13). The reactions ANS has the alternate path ANS2 and TRSP1 has the alternate route formed by TRSP2 and TRSP3 because of which the flux flows through the pathway even in case of deletion of any one of them. Constraining the flux through the TTP reactions to 90% of the flux through those reactions has an effect on the growth rate and flux through the reactions (Supplementary Table S11). In the *E. coli* pathway, reduction in the efficiency of the input and shikimate pathway affects the flux, but not for the reactions ANS, ANS2, TRSP1, TRSP2 and TRSP3 due to the alternate routes, as previously mentioned. Decrease in the efficiency of reactions in the Phe and Tyr section also reduce the flux, out of which the reaction CHORM (chorismate mutase) affects the most, since the flux for the synthesis of Phe and Tyr pass through it. Reduction to 90% of the flux through the reaction has interesting results, for example, when the flux through CHORM is .0072 (.0077 in WT), the flux through the input and Shikimate section is .0084 (.009 in WT), through the Tryptophan section is .0012 (.0013 in WT) and through Phenylalanine is .0039 (.0042 in WT) and Tyrosine is .0033 (.0035 in WT) (Supplementary Table S11; Supplementary Figure S14).

### 2.3 Comparison of network analysis and FBA studies for the TTP pathway

Deletion of hubs can cause a network to lose its structural and functional integrity (Barabási and Oltvai, 2004). Our results (Supplementary Table S4) yielded TTP Network hubs (Nodes having high Degree, Betweenness Centrality, and Closeness Centrality). The reaction deletion studies using FBA analysis also provided a set of the reactions that, when deleted individually, affects the flux through the TTP pathway (Supplementary Table S7 and S12). These two results obtained using different theoretical approaches were compared with each other to find if the Network hubs (of high Degree, BC, and Closeness Centrality) and the essential genes (obtained from FBA reaction deletion analysis) overlap. Table 3 shows the percentage of Degree hubs, BC hubs and Closeness Centrality hubs that were identified using network analysis and also found to be essential reactions for TTP pathway using FBA. Organism specific reactions are those hubs that were identified from the CCN of either *E. coli* or *M. barkeri*. The *Common* hubs are the hubs that were identified to be common across all the 29 organisms that were used in the network analysis. The reactions that are common between Network hubs and the essential reactions from FBA mostly belong to Purine and Pyrimidine biosynthesis, Threonine and Lysine biosynthesis and TTP pathway.

**TABLE 3:** Percentage of Network hub reactions from CCN, which were shown to be essential in the FBA models. Organism specific: hubs of *E. coli* and *M. barkeri*; Common: common hubs of 29 Bacteria and Archaea).

Network	Organism	Degree hubs	Betweenness Centrality hubs	Closeness Centrality hubs
Organism specific	E. coli	0%	19.6%	8.26%
	M. barkeri	62.5%	72.72%	45.45%
Common	E. coli	16.7%	25.50%	24.4%
	M. barkeri	16.7%	41.18%	39%

The Network analysis of the CCN can predict some of the important nodes obtained from FBA analysis. It may be kept in mind that the CCN takes into account only 18 pathways and the reactions present in them, and gives equal weightage to all the reactions and connections. Whereas, in the genome scale FBA, the flux does not flow through all the reactions equally, and hence those reactions and the connections are not reflected in the essential reactions. This indicates that a reduced collection of connected networks can be used to find essential reactions. The list of common hubs across the 29 organisms can be used as a reference list for further studies for finding reactions essential for functioning of TTP pathway and to increase its productivity, since they provide similar result to organism-specific hubs. The list of Network hubs that were shown to be essential by the FBA analysis is given in Supplementary Table S6.

## 3 Discussion

The important role of “context” has been of long-standing empirical and theoretical interest in biological systems because of their multi-scale and interacting modular structures. Understanding context representations and its interaction with functional outcome in behaviour is an area of immense interest to both neurobiologists and in psychology (Rudy, 2009). In an interesting article, the multi-scale and modular structure of metabolic network was analysed to identify the context in which evolutionary processes may occur (Spirin V et al., 2006).

Studies involving molecular interactions of single genes or proteins in the context of their downstream partners and gene context-based modules have been done to evaluate their role in cellular response mechanisms in signalling, amino acids and carbohydrate metabolism pathways (Lan et al., 2013; Bhatt et al., 2018). We started with a general question; *do the topological features (as studied using network analysis) of a metabolic pathway vary when it is embedded in the larger network of other connected pathways, and does this variation affect the pathway function?* We approached to answer this query from a different perspective using two systems biology methods - topological properties (network analysis) and metabolic activity (Flux Balance Analysis) - of the aromatic amino acid biosynthesis (TTP) pathway in many species of Bacteria and Archaea. This pathway consists of quite high energy consuming reactions in the cell. It takes an equivalent of 52, 50 and 74.3 high-energy phosphate bonds for the production of Phenylalanine, Tyrosine and Tryptophan, respectively (Akashi and Gojobori 2002). This energy cost is thus reflected in their usage in the polypeptide chain, and in the metabolic flux passing through the TTP pathway.

The control of the production of aromatic amino acids is traditionally done by means of metabolic engineering in organisms such as *E. coli* and *C. glutamicum* (Katsumata and Ikeda 1993; Ikeda 2006). In these studies, systematic control of genes in the TTP pathway (such as, *aroG, aroF, aroH, anthranilate synthase, pheA etc.*), which respond to the production of the end products, were mutated to increase the production of the aromatic amino acids. Here we have looked at the TTP pathway individually, as well as, when embedded at the larger metabolic network in Bacteria and Archaea. Such studies require various sources of genetic and biochemical information, such as, stoichiometry, structure of reaction pathways and alternative routes of reactions, along with genes and genomes of different organisms. The results presented highlight the fact that functioning of a biochemical reaction in the cell is intimately connected to its “context” (i.e., position of the pathway in the total biochemical network), and the topology of its connectivity to the larger set of reactions - both in the pathway and in the larger biochemical network.

Based on these analyses we are able to arrive at several conclusions. The Network analysis was undertaken to analyse the changes in network properties of TTP pathway reaction network in isolation and in combination with other pathways directly connected to it through sharing of metabolites as incoming or outgoing reactants. The TTP pathway, which is a predominantly linear and a sparse network, shows a low average degree in all organisms. The nodes in the centre of the network possess high Betweenness and high Closeness Centrality values, while the nodes at the extremities show the opposite characteristics. Out of the many pathways that are connected to the TTP pathway, the 17 pathways that were common among the 29 organisms were considered in this study. The network analysis with all connected pathways in all the organisms showed that - changes in the properties of the 15 TTP reaction network nodes not only depended on the topology of the added network, but also on the nodes to which the pathway was added. The Complete Combined Network (CCN), consisting of the TTP pathway and all the 17 connected networks, showed that the properties of the TTP nodes is not the same when considered in the context of the larger connected network. Nodes with low Degree, Betweenness Centrality or Closeness Centrality, either acquire more connections, or by virtue of the new connections that alter the resulting topology, change their network properties, and become hubs in the CCN. The different Degree, Betweenness Centrality and Closeness Centrality hubs were found for the CCN for all the organisms, and the common hubs were ascertained from them. Hence, analyzing pathways in isolation, and in combination with other networks, gives varying properties to the nodes in the network. How these changes in network topology and parameters of the TTP nodes influence the chemical activity leading to end product formations was analyzed using the Flux Balance Analysis.

The Flux Balance Analysis was done to study the flow of metabolites through the metabolic reaction network of the TTP pathway, and to compare it between Bacteria and Archaea, by taking *E. coli* and *M. barkeri* as representatives from the two phyla. The flux through TTP is very low in both the organisms with *M. barkeri* being lower than *E. coli*. *In silico* gene deletion studies of TTP pathway genes showed that fluxes in *M. barkeri* is more resistant to random attack than *E. coli*, due to the presence of isozymes. In both the organisms, the deletion or reduction of efficiency of the gene for Phenylalanine and Tyrosine production greatly affected the overall flux though the network. Deletion of reactions in the whole network showed that many pathways such as, Glycolysis, Histidine metabolism, etc, affect the production of these aromatic amino acids in both the groups of organisms. There are also differences in the pathways, affecting TTP between Bacteria and Archaea, due to their differences in metabolism, such as the Methanogenesis pathway.

A comparison between the network analysis and flux balance analysis of the isolated TTP and CCN of TTP pathways showed that many of the important reaction nodes or “hubs” (in terms of higher network parameters) in the TTP network were common with the essential reactions found by FBA. This points towards identifying a smaller set of reaction steps that can be used for experimental manipulation of the TTP pathway in the cell. This combined Network-FBA approach can be used to predict important reaction steps before attempting any engineering of any pathway for increase or suppression of functionality. Until now, whole genome metabolic networks have been studied by breaking them down into modules using network science (Alcalá-Corona, et al., 2021). This study endeavored to give an integrative view of pathway function and evolution across many prokaryotes, both at a single reaction pathway level, and also when embedded in the larger scheme of biochemical networks. Both the static network approach and the dynamic flux balance analysis offered different perspectives of the same pathway function by arriving at important reaction sets (hubs and essential reactions) that promises to have important applications. Thus, even though the proximate goal of this study (with the PPT pathway as an example) is to understand the contextual role of a specific pathway - in isolation and when embedded in the larger biochemical network of the cell - this approach to study biochemical pathways to understand their systemic properties in the context of biochemical functions inside the cell, may also offer better insight for identifying essential genes, reactions for drug targets, and mutations for improving pathway functions in any organism.

## 4 Materials and methods

### 4.1 Organisms under study

29 Archaeal and Bacterial species (Supplementary Table S1) were considered for the analysis, which consist of Proteobacteria, Halobacteria and Methanomicrobia. Details are given in Supplementary Information.

### 4.2 Division of the pathway

The TTP pathway was broken down into different levels; the lowest level being the individual reactions, thus at individual gene level. The next level was created by dividing the pathway into individual branches or sections that end with the production of important compounds, and the final level was the whole pathway. Figure 1 shows the schematic of a typical TTP pathway. For the ease of understanding and analysis, the TTP pathway is divided into four sections. The first section is the **Input section**, where the genes for the enzymes that catalyze the reactions for the conversion of the initial precursors to 3-dehydroquinate is present. In bacteria, the pathway begins from Erythrose-4-phosphate and Phospho-enol-pyruvate. In many archaea, due to the absence of the oxidative Pentose Phosphate Pathway in several archaeal species (Soderberg 2005), the 3-dehydroquinate necessary for the initial steps of TTP production is produced by DKFP (Porat et al., 2006; Gulko et al., 2014). The second section is the **Shikimate section** of the pathway (Green substrates) which consists of five steps, in which dehydroquinate gets converted to chorismate. The third section is the **Tryptophan section** (Orange substrates), which converts Chorismate, the end product of Shikimate section to Tryptophan. The last section is the **Phenylalanine and Tyrosine section** (Purple and Pink substrates), which consist of genes for the enzymes that sequentially convert Chorismate to Phenylalanine and Tyrosine (Dosselaere and Vanderleyden 2001).

### 4.3 Network analysis

In this analysis, 29 organisms (Bacteria and Archaea) were selected for the study. The details of forming the reaction networks and the list of organisms is given in Supplementary Table S1. The networks were generated using in-house Perl programs. Network parameters such as Degree, Clustering Coefficient, Closeness centrality, Betweenness Centrality (Oldham et al., 2019) were calculated using the igraph package (Csardi and Nepusz 2006) of R (R Core Team 2014). Statistical analysis of the networks was carried out using R and in-house Perl programs.

### 4.4 Flux balance analysis

Flux balance analysis was conducted on *E coli* whole genome model (Feist et al., 2007), as a representative of Bacteria, and, the *M. barkeri* whole genome model (Gonnerman et al., 2013), as a representative of Archaea. The *E. coli* model (iAF1260) consists of 1261 metabolism associated genes, 2382 reactions, and 1668 metabolites. The *M. barkeri* model (iMG746) consists of 746 metabolism associated genes, 815 unique reactions and 718 metabolites. Both the models were simulated in minimal media. The FBA analysis was carried out using Cobrapy .26.0 (Ebrahim et al., 2013), Cobra package for MATLAB and calculations were carried out using in-house python and perl programming. All the data used in this study are available on request.

## Data Availability

The raw data supporting the conclusions of this article will be made available by the authors, without undue reservation.
