# Supplementary material for: A systems level approach to study metabolic networks in prokaryotes with the aromatic amino acid biosynthesis pathway
Source: Front Genet. 2023 Jan 16;13:1084727. doi: 10.3389/fgene.2022.1084727 (PMC9885046; doi:10.3389/fgene.2022.1084727)
Supplement: Supplementary file 1 [file DataSheet1.pdf]

Supplementary information

Supplementary Table S1 *List of organisms for Network analysis*

The list of reactions taking part in TTP, and other pathways connected to TTP, was obtained from KEGG (Kanehisa et al. 2014), MetaCyc (Caspi et al. 2014) and other papers (Herrmann and Weaver 1999; Xie et al. 2003a). The direction of the reactions was obtained from the "mapformula" file in KEGG and MetaCyc databases. The directed reaction networks were created using the reactions as the nodes. A connection or "edge" was established between two reaction nodes if the first reaction (*i*) provided a metabolic product that was used as a reactant in the second reaction (*j*), this constituted a directed edge with direction from *i* node to the *j* node. Self-connections and "currency" metabolites (Ma and Zeng 2003) were removed from the network.

Domain	Organism
**Archaea**	*Halorubrum lacusprofundi* (Hla), *Haloarcula marismortui* (Hma), *Haloterrigena turkmenica* (Htu), *Halorhabdus utahensis* (Hut), *Haloquadratum walsbyi* DSM 16790 (Hwa), *Natronomonas pharaonis* (Nph), *Methanococcus aeolicus* (Mae), *Methanococcus maripaludis* C5 (Mmq), *Methanosarcina acetivorans* (Mac), *Methanosarcina barkeri Fusaro* (Mba), *Methanococcoides burtonii* (Mbu), *Pyrococcus furiosus* DSM 3638 (Pfu), *Sulfolobus islandicus* M.16.4 (Sid)
**Bacteria**	*Bacillus subtilis subsp. subtilis* 168 (Bsu), *Lactococcus lactis subsp. lactis* Il1403 (Lla), *Streptococcus thermophilus* LMG 18311 (Stl), *Clostridium acetobutylicum* ATCC 824 (Cac), *Hungateiclostridium thermocellum* ATCC 27405 (Cth), *Corynebacterium glutamicum* ATCC 13032 (Bielefeld) (Cgb), *Streptomyces coelicolor* (Sco), *Dehalococcoides mccartyi* 195 (Det), Geobacter metallireducens (Gme), *Geobacter sulfurreducens* PCA (Gsu), *Rhodoferax ferrireducens* (Rfr), *Shewanella oneidensis* (Son), *Escherichia coli* K-12 MG1655 (Eco), *Pseudomonas putida* KT2440 (Ppu), *Synechocystis* sp. PCC 6803 (Syn), *Thermotoga maritima* MSB8 (Tma)

Supplementary Table S2 *List of common pathways across the 29 organisms. The pathway names, ids and reactions present in it were taken from KEGG Pathways.*

No:	ID	Pathway
1	**map00400**	Tryptophan, Tyrosine, Phenylalanine biosynthesis (TTP)
2	**map00250**	Alanine, Aspartate and Glutamate metabolism
3	**map00330**	Arginine and Proline metabolism
4	**map00340**	Histidine metabolism
5	**map00970**	Aminoacyl-tRNA biosynthesis
6	**map01120**	Microbial metabolism in diverse environments
7	**map01210**	2-Oxocarboxylic acid metabolism
8	**map01230**	Biosynthesis of amino acids
9	**map00020**	Citrate cycle (TCA cycle)
10	**map01200**	Carbon metabolism
11	**map00010**	Glycolysis / Gluconeogenesis
12	**map00680**	Methane metabolism
13	**map00260**	Glycine, serine and threonine metabolism
14	**map00270**	Cysteine and Methionine metabolism
15	**map00230**	Purine metabolism
16	**map00240**	Pyrimidine metabolism
17	**map00030**	Pentose phosphate pathway
18	**map00051**	Fructose and Mannose metabolism

Supplementary Table S3 *List of common reactions across the 29 organisms.*

No:	ID	Reaction
1	**R02412**	ATP + Shikimate<=> ADP + Shikimate 3-phosphate
2	**R03460**	Phosphoenolpyruvate + Shikimate 3-phosphate <=> Orthophosphate + 5-O-(1-Carboxyvinyl)-3-phosphoshikimate
3	**R01714**	5-O-(1-Carboxyvinyl)-3-phosphoshikimate <=>Chorismate + Orthophosphate
4	**R01715**	Chorismate<=>Prephenate
5	**R01373**	Prephenate<=>Phenylpyruvate + H_2O + CO_2
6	**R00674**	L-Serine + Indole<=> L-Tryptophan + H_2O
7	**R02722**	L-Serine + Indoleglycerol phosphate <=> L-Tryptophan + D-Glyceraldehyde 3-phosphate + H_2O
8	**R02340**	Indoleglycerol phosphate <=>Indole + D-Glyceraldehyde 3-phosphate
9	**R03508**	1-(2-Carboxyphenylamino)-1-deoxy-D-ribulose 5-phosphate <=>Indoleglycerol phosphate + CO_2 + H_2O
10	**R03509**	N-(5-Phospho-D-ribosyl)anthranilate<=> 1-(2-Carboxyphenylamino)-1-deoxy-D-ribulose 5-phosphate
11	**R01073**	N-(5-Phospho-D-ribosyl)anthranilate + Diphosphate<=>Anthranilate + 5-Phospho-alpha-D-ribose 1-diphosphate
12	**R00985**	Chorismate + Ammonia <=>Anthranilate + Pyruvate + H_2O
13	**R00986**	Chorismate + L-Glutamine <=>Anthranilate + Pyruvate + L-Glutamate
14	**R03084**	3-Dehydroquinate <=> 3-Dehydroshikimate + H_2O
15	**R02413**	Shikimate + $NADP^+$ <=> 3-Dehydroshikimate + NADPH + H^+

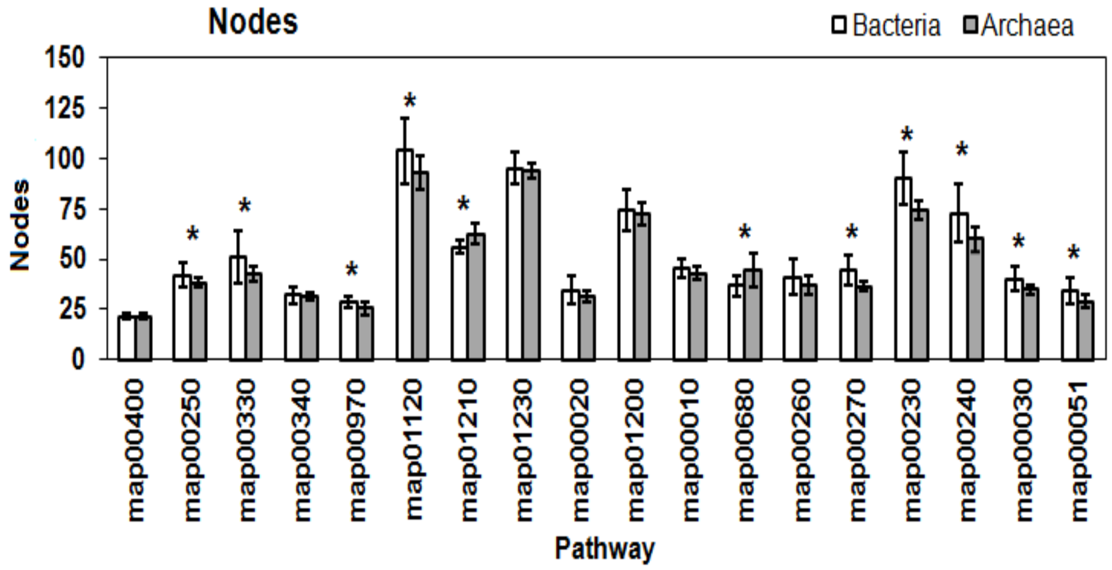

Supplementary Figure S1 *Nodes for each connected network in Bacteria and Archaea.*

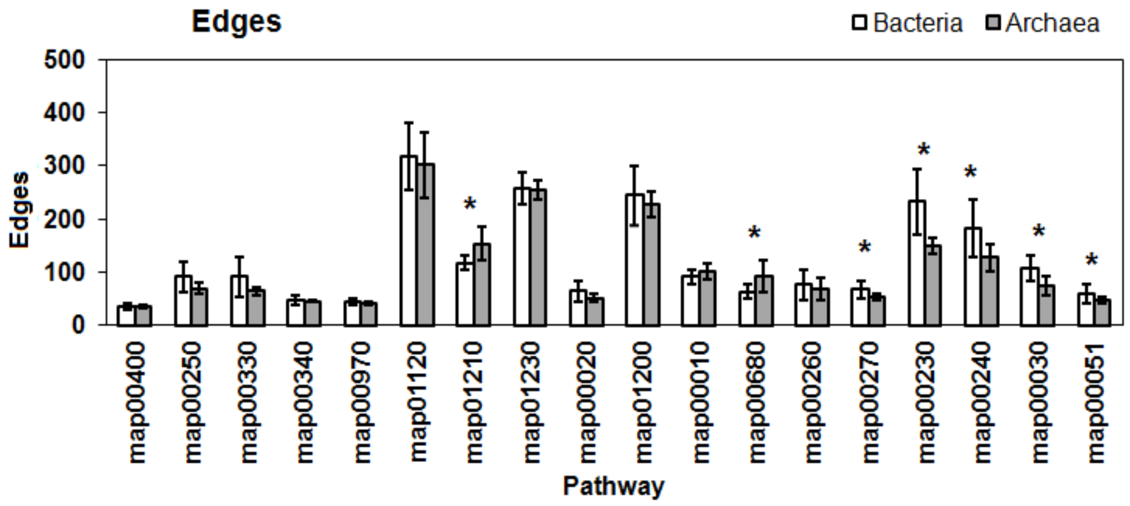

Supplementary Figure S2 *Edges for combined network in Bacteria and Archaea.*

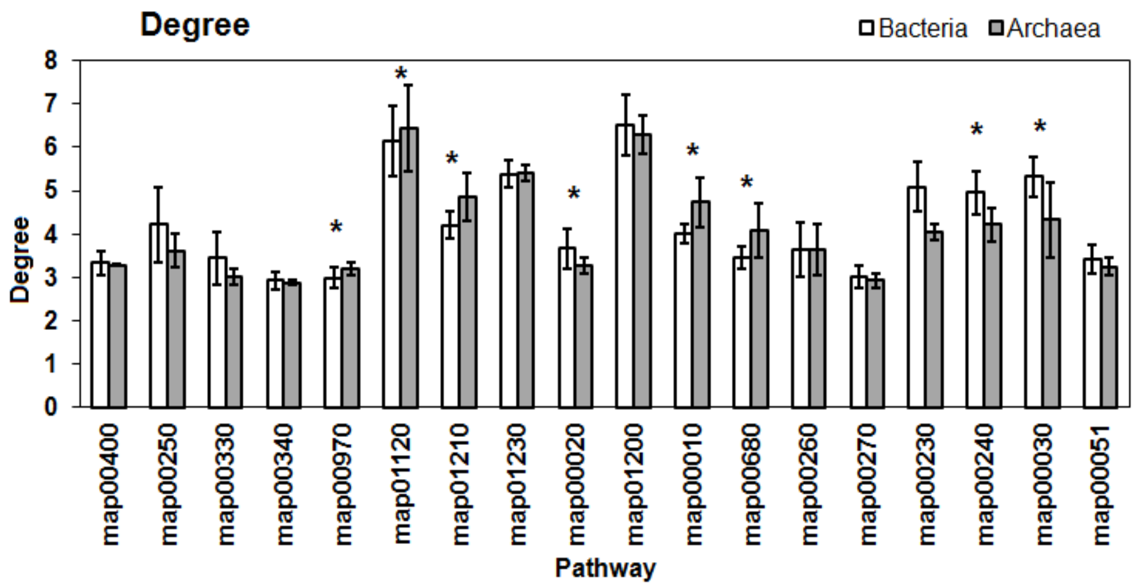

Supplementary Figure S3 *Degree for combined network in Bacteria and Archaea.*

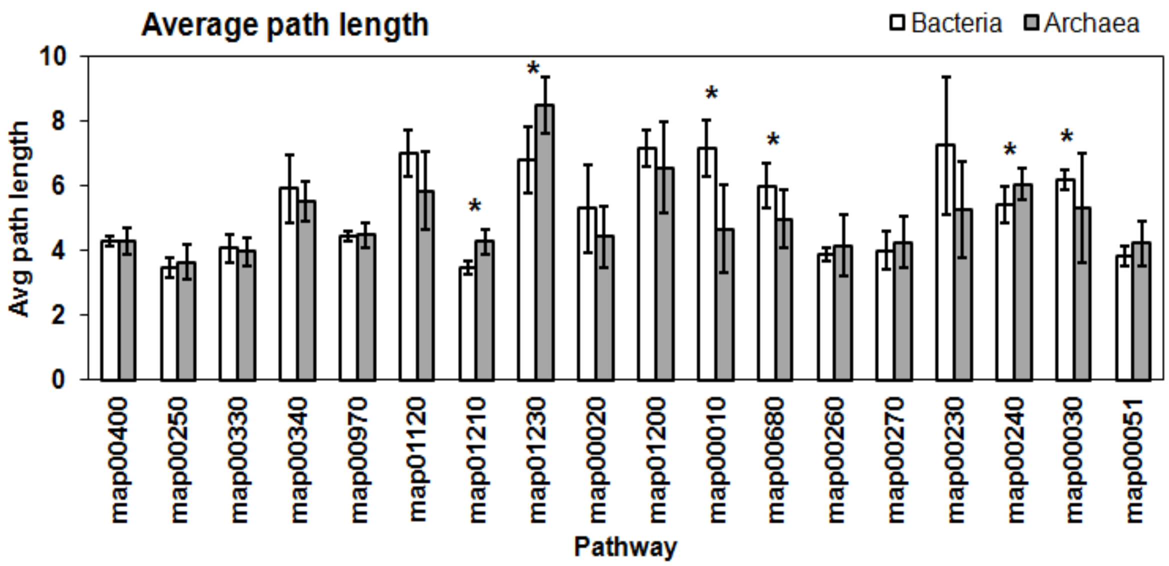

Supplementary Figure S4 *Average path length of the combined networks.*

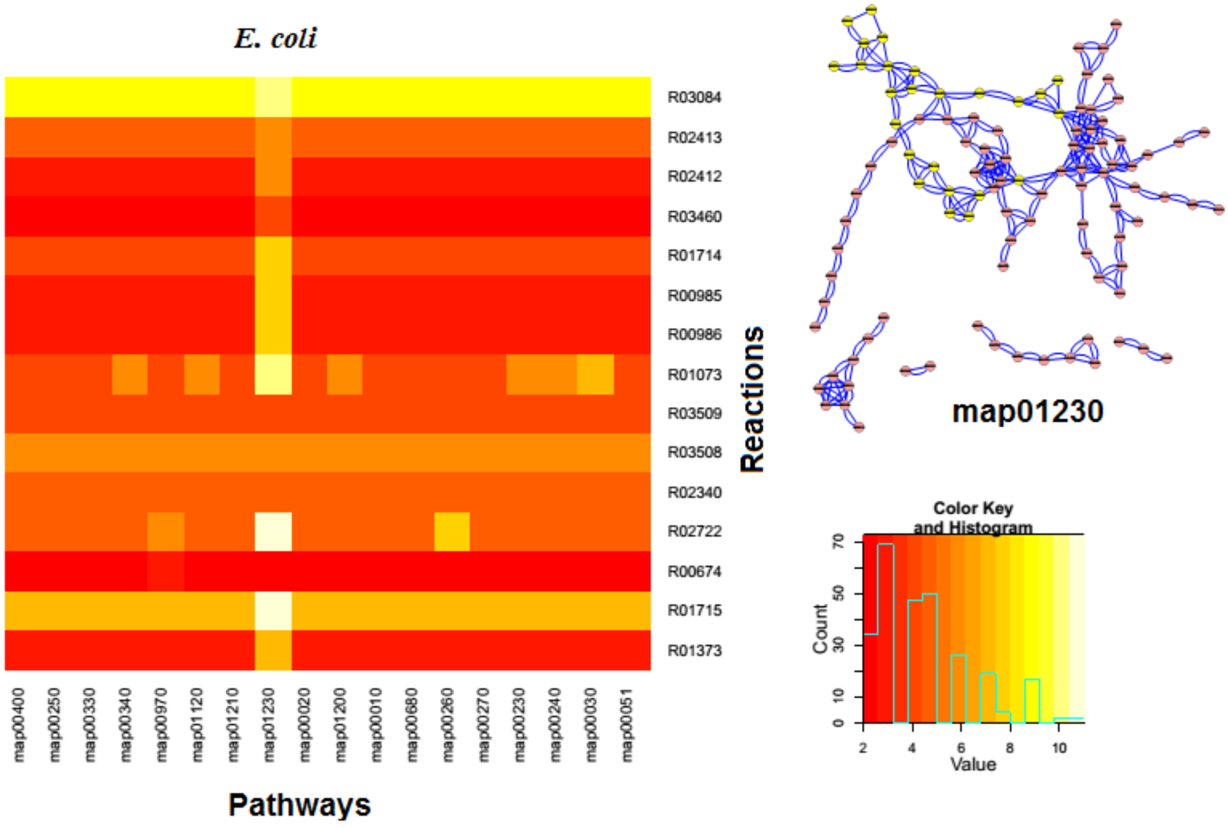

Supplementary Figure S5 *Degree of the reaction nodes present in TTP pathway for different connected networks in bacteria E. coli.*

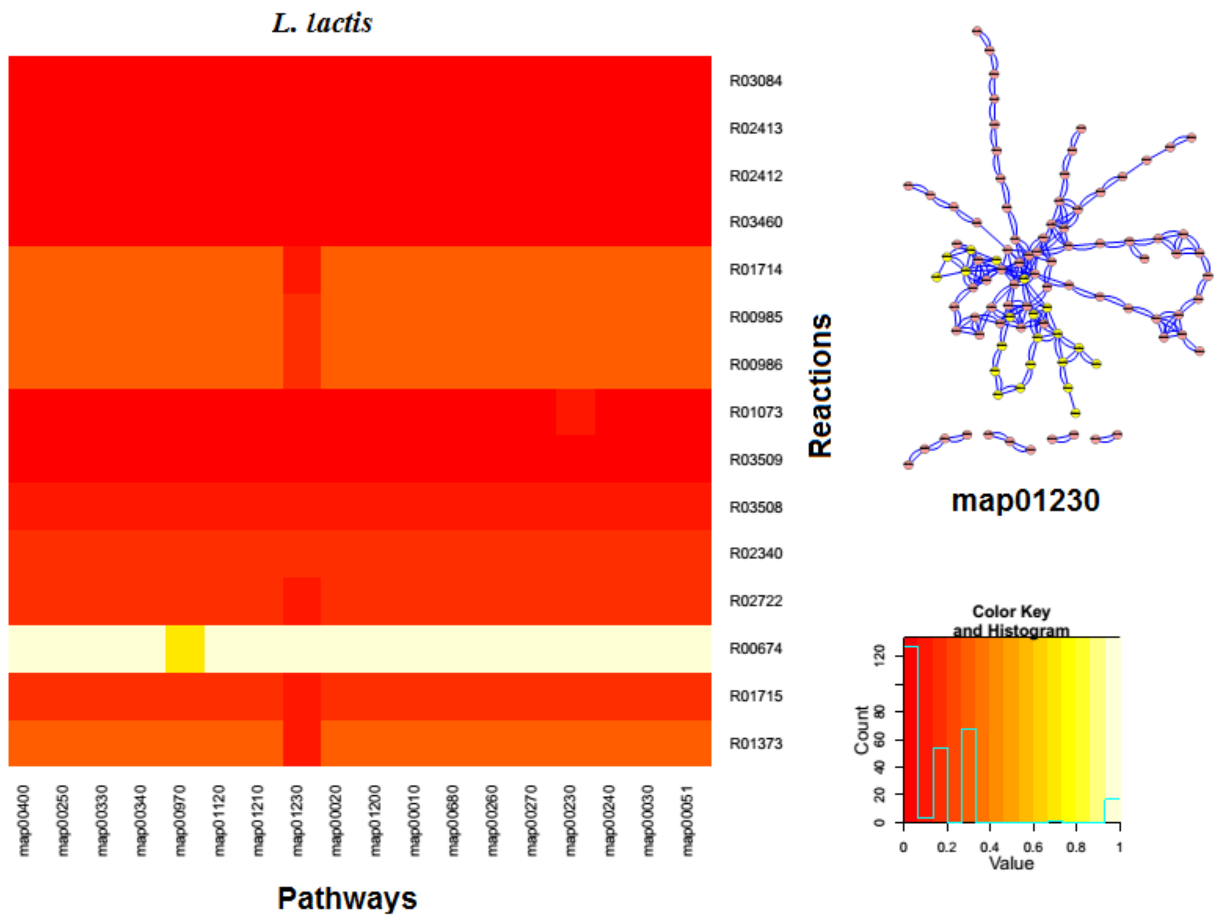

Supplementary Figure S6 *Clustering coefficients of the reactions nodes present in TTP pathway for different connected networks in bacteria L. lactis.*

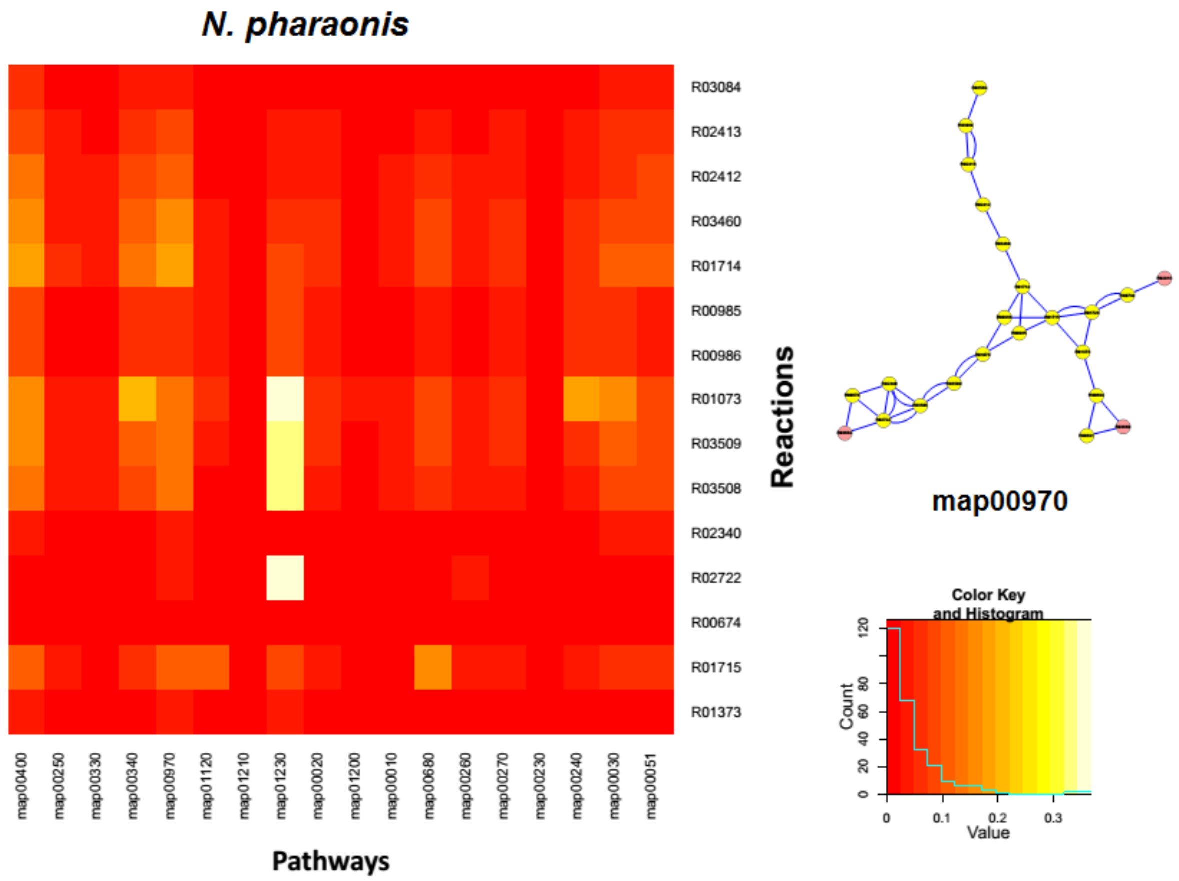

Supplementary Figure S7 *Closeness centrality of the reactions nodes present in TTP pathway for different connected networks in archaea N. pharaonis.*

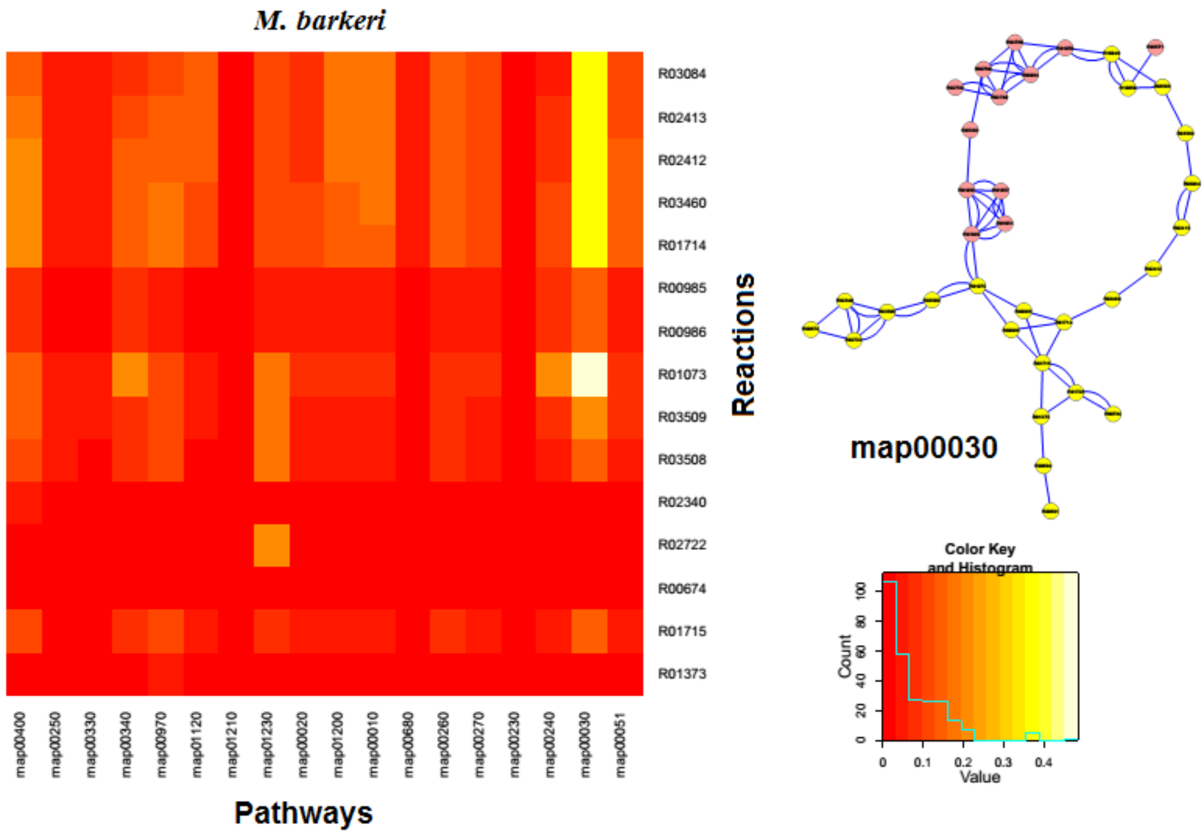

Supplementary Figure S8 *Betweenness Centrality of the reaction nodes present in TTP pathway for different connected networks in Archaea M. barkeri.*

Supplementary Table S4 *List of reaction hubs: D - degree, B – Betweenness, and C Closeness Centrality hubs common to all the 29 organisms. (**Bold italic** - TTP reactions).*

No:	Reaction	Hub			No:	Reaction	Hub		
1	R10147	D	B	C	34	R00378		B	
2	R10052	D	B	C	35	R01466		B	
3	R00253	D	B	C	36	R04441		B	
4	R00441	D	B	C	37	R04440		B	
5	R00442	D	B	C	38	R05071		B	
6	R00443	D	B	C	39	R10170		B	
7	R00435		B	C	40	R02740		B	
8	R01049		B	C	41	R01230		B	
9	R01135		B	C	42	R01231		B	
10	R01083		B	C	43	***R03508***		B	
11	R00158		B	C	44	R03659		B	
12	R01086		B	C	45	***R02412***		B	
13	***R01073***		B	C	46	***R03460***		B	
14	R00127		B	C	47	***R01714***		B	
15	R01773		B	C	48	***R00985***		B	
16	R01775		B	C	49	***R00986***		B	
17	R01954		B	C	50	R01513		B	
18	R02291		B	C	51	R00375		B	
19	***R02722***		B	C	52	***R01373***		B	
20	R00480		B	C	53	R00658			C
21	R00226		B	C	54	R05578			C
22	R00965		B	C	55	R01072			C
23	R03509		B	C	56	R01061			C
24	R01867		B	C	57	R01015			C
25	R01993		B	C	58	R03662			C
26	R01397		B	C	59	R08218			C
27	R01855		B		60	R05577			C
28	R00376		B		61	R01056			C
29	***R01715***		B		62	R05069			C
30	R04559		B		63	R00994			C
31	R04591		B		63	R01518			C
32	***R03084***		B		64	R00720			C
33	***R02413***		B		65	R00575			C
					66	R00426			C

Supplementary Table S5 *List of reactions present in TTP pathway in E. coli and M. barkeri.*

Section	Reaction	KEGG ID	Equation
Input and Shikimate	DKFPASPL		aspsa[c] + dkfp[c] + h[c] + nadh[c] ->dohau[c] + g3p[c] + nad[c]
	DOHDUS	R08569	dohau[c] + h2o[c] + nad[c] ->dohdu[c] + h[c] + nadh[c] + nh4[c]
	DDPA	R01826	e4p[c] + h2o[c] + pep[c] -> 2dda7p[c] + pi[c]
	DHQS	R03083	2dda7p[c] -> 3dhq[c] + pi[c]
	DHQTi	R03084	3dhq[c] -> 3dhsk[c] + h2o[c]
	DHQS2		dohdu[c] -> 3dhq[c]
	DHQD	R03084	3dhq[c] <=> 3dhsk[c] + h2o[c]
	SHK3Dr	R02413	3dhsk[c] + h[c] + nadph[c] <=>nadp[c] + skm[c]
	SHKK	R02412	atp[c] + skm[c] ->adp[c] + h[c] + skm5p[c]
	PSCVT	R03460	pep[c] + skm5p[c] <=> 3psme[c] + pi[c]
	CHORS	R01714	3psme[c] ->chor[c] + pi[c]
Tryptophan	ANS	R00986	chor[c] + gln-L[c] ->anth[c] + glu-L[c] + h[c] + pyr[c]
	ANS2	R00985	chor[c] + nh4[c] ->anth[c] + h2o[c] + h[c] + pyr[c]
	ANPRT	R01073	anth[c] + prpp[c] ->ppi[c] + pran[c]
	PRAIi	R03509	pran[c] -> 2cpr5p[c]
	IGPS	R03508	2cpr5p[c] + h[c] -> 3ig3p[c] + co2[c] + h2o[c]
	TRPS3	R00674	3ig3p[c] -> g3p[c] + indole[c]
	TRPS1	R02722	3ig3p[c] + ser-L[c] -> g3p[c] + h2o[c] + trp-L[c]
	TRPS2	R00674	indole[c] + ser-L[c] <=> h2o[c] + trp-L[c]
	TRPAS2	R00673	indole[c] + nh4[c] + pyr[c] <=> h2o[c] + trp_L[c]
Phenylalanine and Tyrosine	CHORM	R01715	chor[c] ->pphn[c]
	PPNDH	R01373	h[c] + pphn[c] -> co2[c] + h2o[c] + phpyr[c]
	PHETA1	R00694	glu-L[c] + phpyr[c] <=>akg[c] + phe-L[c]
	PPND	R01728	nad[c] + pphn[c] -> 34hpp[c] + co2[c] + nadh[c]
	TYRTA	R00734	34hpp[c] + glu-L[c] <=>akg[c] + tyr-L[c]

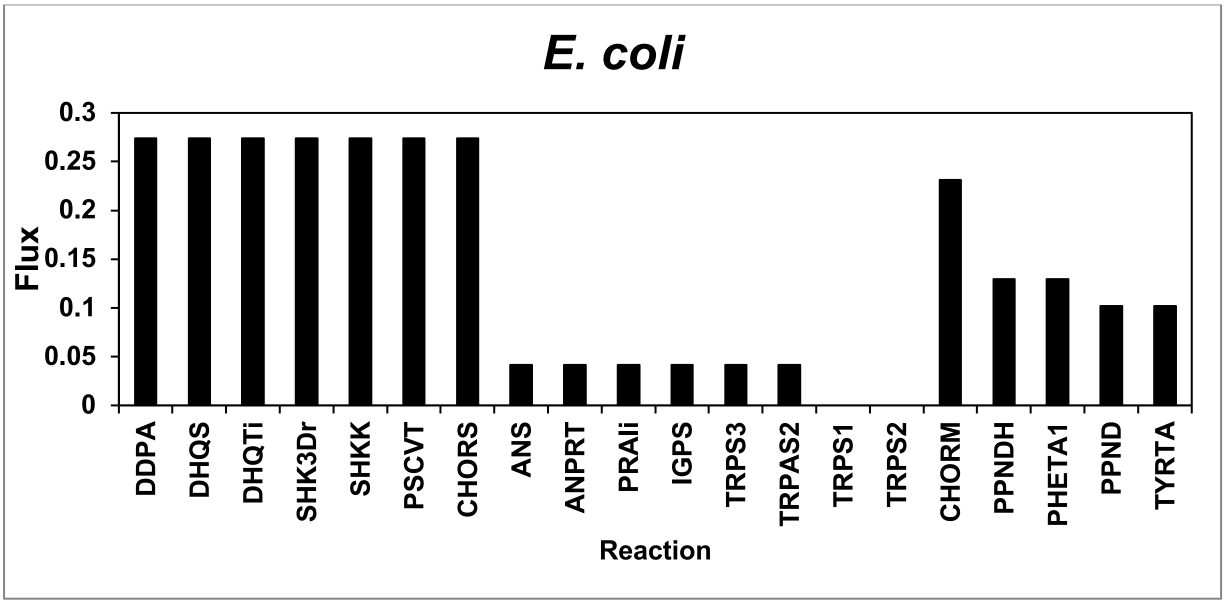

Supplementary Figure S9 *Flux distribution (mmolgDW^{-1}h^{-1}) in the TTP pathway of bacteria E. coli.*

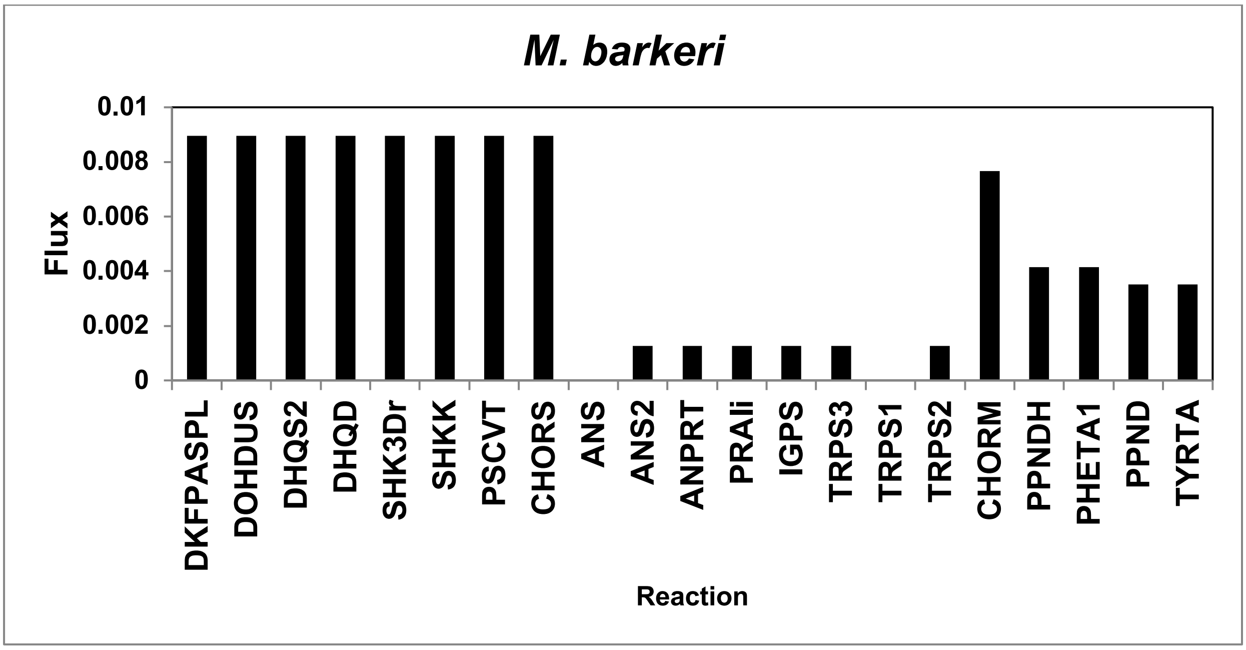

Supplementary Figure S10 *Flux distribution (mmolgDW^{-1}h^{-1}) in the TTP pathway of archaea M. barkeri.*

Supplementary Table S6 *List of common network hubs and essential reactions from FBA. **Bold** – reactions present in TTP pathway.*

FBA model id	Reaction KEGG id	Equation
GLUTRS	R05578	atp[c] + glu-L[c] + trnaglu[c] --> amp[c] + glutrna[c] + ppi[c]
GLNS	R00253	atp + glu-L + nh4 <=>adp + gln-L + h + pi
UMPK	R00158	atp + ump <=>adp + udp
ADSS	R01135	asp-L + gtp + imp <=>dcamp + gdp + 2 h + pi
ASPCT	R01397	asp-L + cbp<=>cbasp + h + pi
DHORTS	R01993	dhor-S + h2o <=>cbasp + h
OMPDC	R00965	h + orot5p <=> co2 + ump
ASAD	R02291	aspsa + nadp + pi <=> 4pasp + h + nadph
ASPK	R00480	asp-L + atp<=> 4pasp + adp
HSDy	R01775	aspsa + h + nadph<=>hom-L + nadp
THRS	R01466	h2o + phom<=> pi + thr-L
**CHORM**	**R01715**	**chor<=>pphn**
**CHORS**	**R01714**	**3psme <=>chor + pi**
DHAD1	R04441	23dhmb[c] --> 3mob[c] + h2o[c]
PRASCS	R04591	5aizc + asp-L + atp<=> 25aics + adp + h + pi
GLUPRT	R01072	gln-L + h2o + prpp<=>glu-L + ppi + pram
PGCD	R01513	3pg + nad<=> 3php + h + nadh
ENO	R00658	2pg <=> h2o + pep
PGM	R01518	2pg <=> 3pg
PRPPS	R01049	atp + r5p <=> amp + h + prpp
RPI	R01056	r5p <=> ru5p-D
ADSL2	R04559	25aics <=>aicar + fum
**ANPRT**	**R01073**	**anth + prpp<=>ppi + pran**
**DHQD**	**R03084**	**3dhq <=> 3dhsk + h2o**
**IGPS**	**R03508**	**2cpr5p + h <=> 3ig3p + co2 + h2o**
**PRAI**	**R03509**	**pran<=> 2cpr5p**
**PSCVT**	**R03460**	**pep + skm5p <=> 3psme + pi**
**SHK3D**	**R02413**	**3dhsk + h + nadph<=>nadp + skm**
**SHKK**	**R02412**	**atp + skm<=>adp + h + skm5p**

Supplementary Table S7 *Pathways that affect TTP production in E.coli.*

Alanine and Aspartate Metabolism	Cell Envelope Biosynthesis
Unassigned	Membrane Lipid Metabolism
Alternate Carbon Metabolism	Lipopolysaccharide Biosynthesis Recycling
Folate Metabolism	Citric Acid Cycle
Arginine and Proline Metabolism	Glutamate Metabolism
Threonine and Lysine Metabolism	ValineLeucine and Isoleucine Metabolism
Cofactor and Prosthetic Group Biosynthesis	Cysteine Metabolism
Inorganic Ion Transport and Metabolism	Murein metabolism
Methionine Metabolism	Glycerophospholipid Metabolism
Nucleotide Salvage Pathway	Histidine Metabolism
Transport Inner Membrane	Oxidative Phosphorylation
Transport Outer Membrane Porin	Purine and Pyrimidine Biosynthesis

Supplementary Table S8 *Flux in the TTP pathway after single gene deletion in E. coli. The rest of the reactions have no flux passing through them.*

	Wild type	Deleted Reaction			
		TRPS3	TRPAS2	TRPS1	TRPS2
DDPA	0.274	0.274	0.274	0.274	0.274
DHQS	0.274	0.274	0.274	0.274	0.274
DHQTi	0.274	0.274	0.274	0.274	0.274
SHK3Dr	0.274	0.274	0.274	0.274	0.274
SHKK	0.274	0.274	0.274	0.274	0.274
PSCVT	0.274	0.274	0.274	0.274	0.274
CHORS	0.274	0.274	0.274	0.274	0.274
ANS	0.042	0.042	0.042	0.042	0.042
ANPRT	0.042	0.042	0.042	0.042	0.042
PRAIi	0.042	0.042	0.042	0.042	0.042
IGPS	0.042	0.042	0.042	0.042	0.042
TRPS3	0.042	0	0.042	0.042	0.042
TRPAS2	-0.042	0	0	-0.042	-0.042
TRPS1	0	0.042	0	0	0
TRPS2	0	0	0.042	0	0
CHORM	0.231	0.231	0.231	0.231	0.231
PPNDH	0.13	0.13	0.13	0.13	0.13
PHETA1	-0.13	-0.13	-0.13	-0.13	-0.13
PPND	0.102	0.102	0.102	0.102	0.102
TYRTA	-0.102	-0.102	-0.102	-0.102	-0.102

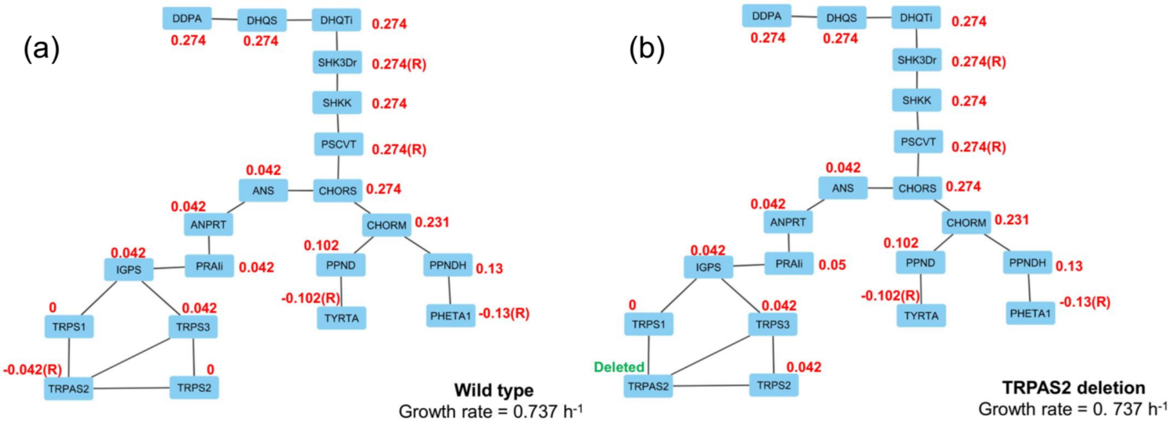

Supplementary Figure S11 *Comparison between the flux passing through TTP pathway in (a) Wild type flux and (b) TRPAS2 deletion in E. coli.*

Supplementary Table S9 *Flux in the TTP pathway after individual reactions are constrained to 90% in E. coli.*

		Reaction constrained to 90% of WT flux																			
	Wild Type	DDPA	DHQS	DHQTi	SHK3Dr	SHKK	PSCVT	CHORS	ANS	ANPRT	PRAIi	IGPS	TRPS3	TRPAS2	TRPS1	TRPS2	CHORM	PPNDH	PHETA1	PPND	TYRTA
DDPA	0.274	0.247	0.247	0.247	0.247	0.247	0.247	0.247	0.247	0.247	0.247	0.247	0.274	0.274	0.274	0.274	0.246	0.247	0.247	0.247	0.247
DHQS	0.274	0.247	0.247	0.247	0.247	0.247	0.247	0.247	0.247	0.247	0.247	0.247	0.274	0.274	0.274	0.274	0.246	0.247	0.247	0.247	0.247
DHQTi	0.274	0.247	0.247	0.247	0.247	0.247	0.247	0.247	0.247	0.247	0.247	0.247	0.274	0.274	0.274	0.274	0.246	0.247	0.247	0.247	0.247
SHK3Dr	0.274	0.247	0.247	0.247	0.247	0.247	0.247	0.247	0.247	0.247	0.247	0.247	0.274	0.274	0.274	0.274	0.246	0.247	0.247	0.247	0.247
SHKK	0.274	0.247	0.247	0.247	0.247	0.247	0.247	0.247	0.247	0.247	0.247	0.247	0.274	0.274	0.274	0.274	0.246	0.247	0.247	0.247	0.247
PSCVT	0.274	0.247	0.247	0.247	0.247	0.247	0.247	0.247	0.247	0.247	0.247	0.247	0.274	0.274	0.274	0.274	0.246	0.247	0.247	0.247	0.247
CHORS	0.274	0.247	0.247	0.247	0.247	0.247	0.247	0.247	0.247	0.247	0.247	0.247	0.274	0.274	0.274	0.274	0.246	0.247	0.247	0.247	0.247
ANS	0.042	0.038	0.038	0.038	0.038	0.038	0.038	0.038	0.038	0.038	0.038	0.038	0.042	0.042	0.042	0.042	0.038	0.038	0.038	0.038	0.038
ANPRT	0.042	0.038	0.038	0.038	0.038	0.038	0.038	0.038	0.038	0.038	0.038	0.038	0.042	0.042	0.042	0.042	0.038	0.038	0.038	0.038	0.038
PRAIi	0.042	0.038	0.038	0.038	0.038	0.038	0.038	0.038	0.038	0.038	0.038	0.038	0.042	0.042	0.042	0.042	0.038	0.038	0.038	0.038	0.038
IGPS	0.042	0.038	0.038	0.038	0.038	0.038	0.038	0.038	0.038	0.038	0.038	0.038	0.042	0.042	0.042	0.042	0.038	0.038	0.038	0.038	0.038
TRPS3	0.042	0.038	0.038	0.038	0.038	0.038	0.038	0.038	0.038	0.038	0.038	0.038	0.038	0.042	0.042	0.042	0.038	0.038	0.038	0.038	0.038
TRPAS2	-0.042	-0.038	-0.038	-0.038	-0.038	-0.038	-0.038	-0.038	-0.038	-0.038	-0.038	-0.038	-0.038	-0.038	-0.042	-0.042	-0.038	-0.038	-0.038	-0.038	-0.038
TRPS1	0	0	0	0	0	0	0	0	0	0	0	0	0.004	0	0	0	0	0	0	0	0
TRPS2	0	0	0	0	0	0	0	0	0	0	0	0	0	0.004	0	0	0	0	0	0	0
CHORM	0.231	0.208	0.208	0.208	0.208	0.208	0.208	0.208	0.209	0.209	0.209	0.209	0.231	0.231	0.231	0.231	0.208	0.209	0.209	0.209	0.209
PPNDH	0.13	0.117	0.117	0.117	0.117	0.117	0.117	0.117	0.117	0.117	0.117	0.117	0.13	0.13	0.13	0.13	0.116	0.117	0.117	0.117	0.117
PHETA1	-0.13	-0.117	-0.117	-0.117	-0.117	-0.117	-0.117	-0.117	-0.117	-0.117	-0.117	-0.117	-0.13	-0.13	-0.13	-0.13	-0.116	-0.117	-0.117	-0.117	-0.117
PPND	0.102	0.092	0.092	0.092	0.092	0.092	0.092	0.092	0.092	0.092	0.092	0.092	0.102	0.102	0.102	0.102	0.091	0.092	0.092	0.092	0.092
TYRTA	-0.102	-0.092	-0.092	-0.092	-0.092	-0.092	-0.092	-0.092	-0.092	-0.092	-0.092	-0.092	-0.102	-0.102	-0.102	-0.102	-0.091	-0.092	-0.092	-0.092	-0.092

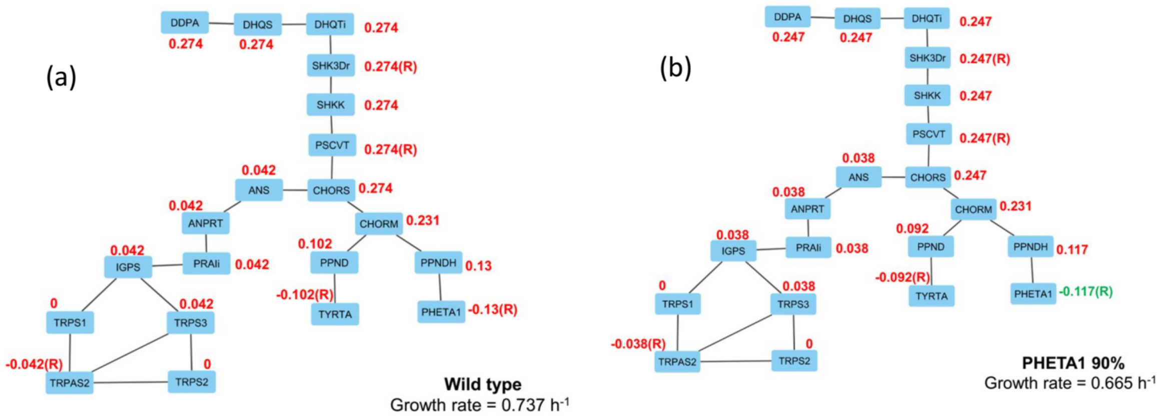

Supplementary Figure S12 *Comparison between the flux passing through TTP pathway in E. coli (a) Wild type flux and (b) PHETA1 constrained to 90% of the wild type flux.*

Supplementary Table S10 *Flux in the TTP pathway after single gene deletion in M. barkeri. The rest of the reactions have no flux passing through them.*

	Wild type	Deleted reactions				
		ANS	ANS2	TRPS3	TRPS1	TRPS2
DKFPASPL	0.009	0.009	0.0089	0.009	0.009	0.009
DOHDUS	0.009	0.009	0.0089	0.009	0.009	0.009
DHQS2	0.009	0.009	0.0089	0.009	0.009	0.009
DHQD	0.009	0.009	0.0089	0.009	0.009	0.009
SHK3Dr	0.009	0.009	0.0089	0.009	0.009	0.009
SHKK	0.009	0.009	0.0089	0.009	0.009	0.009
PSCVT	0.009	0.009	0.0089	0.009	0.009	0.009
CHORS	0.009	0.009	0.0089	0.009	0.009	0.009
ANS	0	0	0.0013	0	0	0
ANS2	0.0013	0.0013	0	0.0013	0.0013	0.0013
ANPRT	0.0013	0.0013	0.0013	0.0013	0.0013	0.0013
PRAIi	0.0013	0.0013	0.0013	0.0013	0.0013	0.0013
IGPS	0.0013	0.0013	0.0013	0.0013	0.0013	0.0013
TRPS3	0	0	0	0	0.0013	0
TRPS1	0.0013	0.0013	0.0013	0.0013	0	0.0013
TRPS2	0	0	0	0	0.0013	0
CHORM	0.0077	0.0077	0.0077	0.0077	0.0077	0.0077
PPNDH	0.0042	0.0042	0.0042	0.0042	0.0042	0.0042
PHETA1	-0.0042	-0.0042	-0.0042	-0.0042	-0.0042	-0.0042
PPND	0.0035	0.0035	0.0035	0.0035	0.0035	0.0035
TYRTA	-0.0035	-0.0035	-0.0035	-0.0035	-0.0035	-0.0035

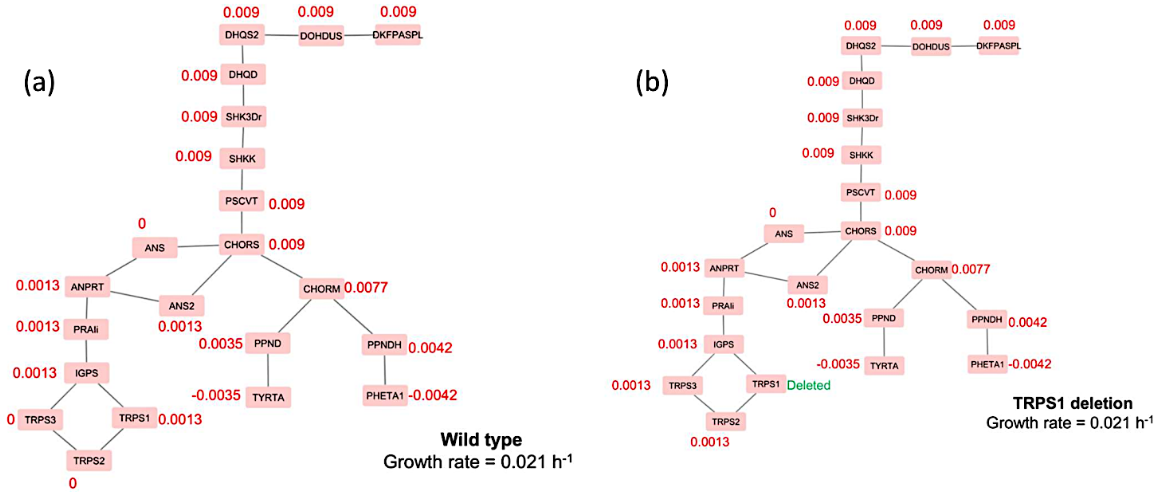

Supplementary Figure S13 *Comparison between the flux passing through TTP pathway in (a) Wild type flux and (b) TRPAS2 deletion in M. barkeri*

Supplementary Table S11 *Flux in the TTP pathway in M. barkeri after flux through individual reactions is constrained to 90%.*

		Reaction constrained to 90% of the WT flux																				
	Wild Type	DKFPASPL	DOHDUS	DHQS2	DHQD	SHK3Dr	SHKK	PSCVT	CHORS	ANS	ANS2	ANPRT	PRAIi	IGPS	TRPS3	TRPS1	TRPS2	CHORM	PPNDH	PHETA1	PPND	TYRTA
DKFPASPL	0.009	0.0081	0.0081	0.0081	0.0081	0.0081	0.0081	0.0081	0.0081	0.009	0.0089	0.0063	0.0063	0.0063	0.009	0.009	0.009	0.0084	0.0078	0.0078	0.009	0.009
DOHDUS	0.009	0.0081	0.0081	0.0081	0.0081	0.0081	0.0081	0.0081	0.0081	0.009	0.0089	0.0063	0.0063	0.0063	0.009	0.009	0.009	0.0084	0.0078	0.0078	0.009	0.009
DHQS2	0.009	0.0081	0.0081	0.0081	0.0081	0.0081	0.0081	0.0081	0.0081	0.009	0.0089	0.0063	0.0063	0.0063	0.009	0.009	0.009	0.0084	0.0078	0.0078	0.009	0.009
DHQD	0.009	0.0081	0.0081	0.0081	0.0081	0.0081	0.0081	0.0081	0.0081	0.009	0.0089	0.0063	0.0063	0.0063	0.009	0.009	0.009	0.0084	0.0078	0.0078	0.009	0.009
SHK3Dr	0.009	0.0081	0.0081	0.0081	0.0081	0.0081	0.0081	0.0081	0.0081	0.009	0.0089	0.0063	0.0063	0.0063	0.009	0.009	0.009	0.0084	0.0078	0.0078	0.009	0.009
SHKK	0.009	0.0081	0.0081	0.0081	0.0081	0.0081	0.0081	0.0081	0.0081	0.009	0.0089	0.0063	0.0063	0.0063	0.009	0.009	0.009	0.0084	0.0078	0.0078	0.009	0.009
PSCVT	0.009	0.0081	0.0081	0.0081	0.0081	0.0081	0.0081	0.0081	0.0081	0.009	0.0089	0.0063	0.0063	0.0063	0.009	0.009	0.009	0.0084	0.0078	0.0078	0.009	0.009
CHORS	0.009	0.0081	0.0081	0.0081	0.0081	0.0081	0.0081	0.0081	0.0081	0.009	0.0089	0.0063	0.0063	0.0063	0.009	0.009	0.009	0.0084	0.0078	0.0078	0.009	0.009
ANS	0	0	0	0	0	0	0	0	0	0	0.0004	0	0	0	0	0	0	0	0	0	0	0
ANS2	0.0013	0.0012	0.0012	0.0012	0.0012	0.0012	0.0012	0.0012	0.0012	0.0013	0.0009	0.0009	0.0009	0.0009	0.0013	0.0013	0.0013	0.0012	0.0011	0.0011	0.0013	0.0013
ANPRT	0.0013	0.0012	0.0012	0.0012	0.0012	0.0012	0.0012	0.0012	0.0012	0.0013	0.0013	0.0009	0.0009	0.0009	0.0013	0.0013	0.0013	0.0012	0.0011	0.0011	0.0013	0.0013
PRAIi	0.0013	0.0012	0.0012	0.0012	0.0012	0.0012	0.0012	0.0012	0.0012	0.0013	0.0013	0.0009	0.0009	0.0009	0.0013	0.0013	0.0013	0.0012	0.0011	0.0011	0.0013	0.0013
IGPS	0.0013	0.0012	0.0012	0.0012	0.0012	0.0012	0.0012	0.0012	0.0012	0.0013	0.0013	0.0009	0.0009	0.0009	0.0013	0.0013	0.0013	0.0012	0.0011	0.0011	0.0013	0.0013
TRPS3	0	0	0	0	0	0	0	0	0	0	0	0	0	0	0	0.0004	0	0	0	0	0	0
TRPS1	0.0013	0.0012	0.0012	0.0012	0.0012	0.0012	0.0012	0.0012	0.0012	0.0013	0.0013	0.0009	0.0009	0.0009	0.0013	0.0009	0.0013	0.0012	0.0011	0.0011	0.0013	0.0013
TRPS2	0	0	0	0	0	0	0	0	0	0	0	0	0	0	0	0.0004	0	0	0	0	0	0
CHORM	0.0077	0.0069	0.0069	0.0069	0.0069	0.0069	0.0069	0.0069	0.0069	0.0077	0.0077	0.0054	0.0054	0.0054	0.0077	0.0077	0.0077	0.0072	0.0067	0.0067	0.0078	0.0078
PPNDH	0.0042	0.0038	0.0038	0.0038	0.0038	0.0038	0.0038	0.0038	0.0038	0.0042	0.0042	0.0029	0.0029	0.0029	0.0042	0.0042	0.0042	0.0039	0.0036	0.0036	0.0042	0.0042
PHETA1	-0.0042	-0.0038	-0.0038	-0.0038	-0.0038	-0.0038	-0.0038	-0.0038	-0.0038	-0.0042	-0.0042	-0.0029	-0.0029	-0.0029	-0.0042	-0.0042	-0.0042	-0.0039	-0.0036	-0.0036	-0.0042	-0.0042

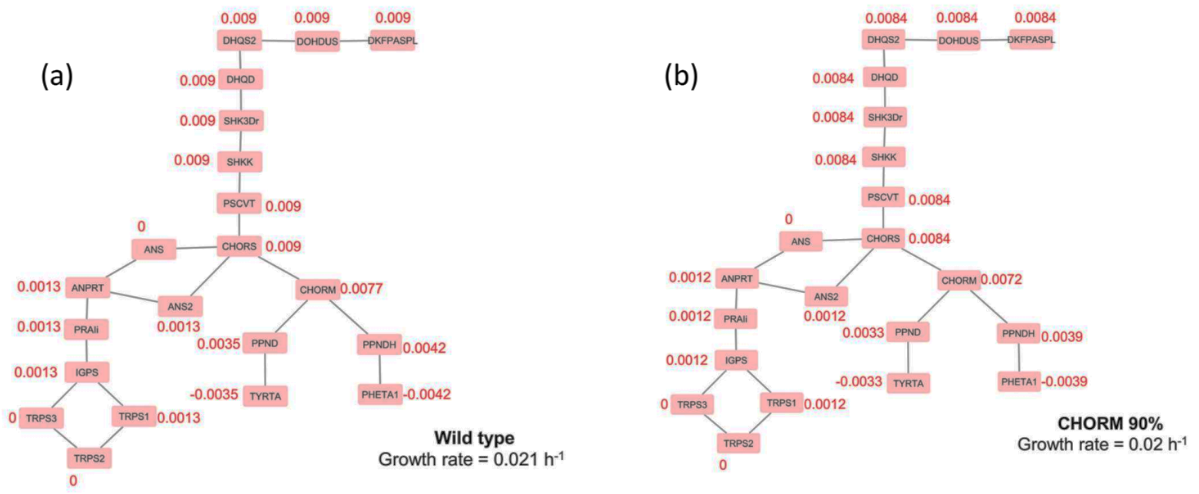

Supplementary Figure S14 *Comparison between the flux passing through TTP pathway in M. barkeri (a) Wild type flux and (b) CHROM constrained to 90% of the wild type flux.*

Supplementary Table S12 *Pathways that affect TTP production in M. barkeri*

**Alanine and Aspartate Metabolism**	**Histidine Metabolism**
**Amino Acid Metabolism**	**Lipid Cell Wall Metabolism**
**Arginine and Proline Metabolism**	**Methanofuran B Biosynthesis**
**Central Metabolism**	**Methanogenesis**
**Citric Acid Cycle**	**Methionine Metabolism**
**Coenzyme A Biosynthesis**	**Nucleotide Metabolism**
**Coenzyme B Biosynthesis**	**Threonine and Lysine Metabolism**
**Glutamate metabolism**	**Transport**
**Glutamine Metabolism**	**Valine Leucine and Isoleucine Metabolism**
**Glycine and Serine Metabolism**	**Vitamins Cofactor Biosynthesis**
**Glycolysis/Gluconeogenesis**	

Bibliography

Caspi R, Altman T, Billington R, *et al* (2014) The MetaCyc database of metabolic pathways and enzymes and the BioCyc collection of Pathway/Genome Databases. Nucleic Acids Res 42:D459-471. doi:10.1093/nar/gkt1103

Herrmann KM, Weaver LM (1999) The Shikimate Pathway. Annu Rev Plant Physiol Plant Mol Biol 50:473–503

Kanehisa M, Goto S, Sato Y, *et al* (2014) Data, information, knowledge and principle: Back to metabolism in KEGG. Nucleic Acids Res 42:199–205. doi:10.1093/nar/gkt1076

Ma, H., and Zeng, A. (2003). Reconstruction of metabolic networks from genome data and analysis of their global structure for various organisms. Bioinformatics 19:270–277. doi:10.1093/bioinformatics/19.2.270

Xie G, Keyhani NO, Bonner C, Jensen R (2003a) Ancient origin of the tryptophan operon and the dynamics of evolutionary change. Microbiol Mol Biol Rev 67:303–342. doi:10.1128/MMBR.67.3.303-342.2003
